# Targeting the NAD^+^ Salvage Pathway Blocks Metabolic Recovery and Enhances β-Lapachone Toxicity in NQO1-Expressing Glioblastoma Cells

**DOI:** 10.1158/2767-9764.CRC-26-0275

**Published:** 2026-08-03

**Authors:** Bruce Chang-Gu, Tuvshintugs Baljinnyam, Mark L. Sowers, Rahul Dilawari, Jason Herring, Lawrence Sowers

**Affiliations:** 1 University of Texas Medical Branch at Galveston, Galveston, Texas.; 2 MD-PhD Combined Degree Program, University of Texas Medical Branch, Galveston, Texas.; 3 Department of Pharmacology and Toxicology, University of Texas Medical Branch, Galveston, Texas.

## Abstract

Glioblastoma (GBM) is a highly heterogeneous tumor, with some cell populations overexpressing genes that give them a survival advantage. One such gene is NAD(P)H quinone oxidoreductase 1 (*NQO1*), which protects cells from oxidative stress. Although NQO1 overexpression shields cells from endogenous oxidants, the enzyme can also bioactivate certain compounds, including the naphthoquinone β-lapachone (β-lap). This activation triggers repeated cycles of oxidative stress that ultimately drive cell death. In this study, we investigated whether NQO1 overexpression could be exploited as a selective vulnerability to induce toxicity in GBM cells. NQO1 expression status in the U87 GBM cell line, multiple patient-derived GBM cell lines, and normal human astrocytes (NHA) was evaluated. Dose–response studies, NAD^+^ quantification, and immunoblot analysis were utilized to evaluate the link between NAD^+^ synthesis and β-lap toxicity. We demonstrate that NQO1 is highly expressed in multiple GBM cell lines and β-lap induces selective cytotoxicity in these cells while sparing low NQO1-expressing cells including NHAs. β-lap induces acute NAD^+^ depletion and DNA damage. However, high NQO1-expressing GBM cells may regenerate NAD^+^ and evade β-lap toxicity. We identified the NAD^+^ salvage pathway to be the primary pathway responsible for maintaining NAD^+^ levels in GBM. We demonstrate that targeting this pathway with the nicotinamide phosphoribosyltransferase (NAMPT) inhibitor FK866 prevents NAD^+^ regeneration after β-lap exposure and enhances β-lap cytotoxicity in NQO1-expressing GBM. Altered NAD^+^ metabolism in GBM represents a potential metabolic vulnerability. Our results suggest that targeting NQO1-expressing GBM with NQO1-bioactivatable compounds in combination with NAMPT inhibitors is a promising therapeutic strategy for the treatment of GBM.

**Significance:**

NAD^+^ metabolism is altered in GBM. However, there are limited therapeutic options for targeting this metabolic vulnerability. This study identifies NQO1+ GBM as highly susceptible to β-lap–induced NAD^+^ depletion and cell death. Blocking the NAD^+^ salvage pathway prevents metabolic recovery in GBM and increases β-lap toxicity in NQO1+ cells.

## Introduction

Glioblastoma (GBM) is the most common and aggressive primary adult central nervous system (CNS) malignancy. The current standard of care has remained unchanged in the past 2 decades and consists of maximal surgical resection followed by concurrent treatment with the chemotherapy agent temozolomide and radiotherapy (1). Despite treatment, the median survival remains at 15 months after initial diagnosis (1–4).

Previous transcriptomic studies have differentiated GBM cells into distinct molecular phenotypes including proneural, mesenchymal, and classical subtypes (5–7). These studies have improved our understanding of GBM biology and have facilitated the identification of novel therapeutic targets in GBM thought to confer multitherapy resistance (8). We and others have demonstrated that NAD^+^ metabolism is altered in GBM and may be a potential target for the treatment of GBM (9–11). Besides its role in redox reactions in energy-producing pathways (12, 13), NAD^+^ is also the primary substrate for numerous ADP-ribose transferases (9, 12, 13) including poly (ADP-ribose) polymerase (PARP) which participates in DNA damage repair and its activity is thought to confer chemotherapy resistance in GBM (9, 14).

Here, we demonstrate targeting NAD(P)H quinone oxidoreductase (NQO1) to deplete NAD^+^ stores in GBM. NQO1 plays an important role in managing oxidative stress through 2e^−^ reduction of endogenous quinones and has previously been identified to be upregulated in many malignant cancers (11, 15, 16). In GBM, elevated NQO1 expression has been associated with increased cell proliferation, radiotherapy resistance, and decreased patient survival (15). Elevated NQO1 expression in cancer cells may be exploited by various NQO1-bioactivatable naphthoquinones including plumbagin, lapachol, and β-lapachone (β-lap; refs. 17, 18). Naphthoquinones generate reactive oxidative species, leading to oxidative stress, PARP-mediated NAD^+^ depletion, and eventual cell death in NQO1-expressing cancer cells (11, 19). Mechanistically, β-lap induces toxicity through NAD^+^ depletion (20), and additional agents targeting NAD^+^ synthesis have previously been investigated to improve β-lap’s toxicity toward other tumors (16, 21, 22).

In this study, we use a panel of previously characterized patient-derived xenograft (PDX) GBM cell lines (23) to show that NQO1 is selectively overexpressed in a subset of GBM but absent in normal human astrocytes (NHA). Mechanistically, we show that targeting NQO1 with the naphthoquinone β-lap induces acute DNA damage, toxicity, and NAD^+^ depletion in NQO1-expressing GBM. We also demonstrate synergistic combinations of β-lap and the NAD^+^ salvage inhibitor FK866 to significantly increase β-lap toxicity in NQO1-expressing GBM by preventing regeneration of NAD^+^. Our findings highlight NQO1 expression as a metabolic vulnerability in GBM and targeting NQO1-expressing GBM with NQO1-activated compounds as a strategy to target metabolically distinct tumor populations.

## Materials and Methods

### Chemicals and reagents

β-lap (cat. #L2037), DMSO (cat. #276855), trypan blue dye (0.4%; cat. #T8154), phenazine methosulfate (cat. #P9625), 3-(4,5-di methyl thiazol-2-yl)-2,5-diphenyltetrazolium bromide (MTT; cat. #M2128), *Saccharomyces cerevisiae* alcohol dehydrogenase (EC1.1.1.1; cat. #A7011), bicinchoninic acid (BCA; cat. #71285-3), Immobilon-P polyvinylidene difluoride membrane (cat. #IPFL00010), and Tween 20 (cat. #P9416) were purchased from Sigma-Aldrich. Tris base (cat. #BP152), 10× Tris-buffered saline (TBS; cat. #BP2471-1), 200-proof ethanol (cat. #AC615090040), concentrated HCl (cat. #A144-212), L-methionine (cat. #BP388), and sodium bicarbonate (cat. #BP328) were purchased from Thermo Fisher Scientific. Furthermore, 4× Laemmli buffer (cat. #1610747), 4% to 15% Mini-PROTEAN Tris-Glycine eXtended (TGX) gel (cat. #4561083), and blotting-grade blocker (cat. #1706404) were purchased from Bio-Rad. Tris-HCl (cat. #H5123), NAD^+^ standard (cat. #E2ND EFND), and phosphate-buffered saline (PBS; cat. #21-031-CM) were purchased from Promega, BioAssay Systems, and Corning, respectively.

### Cell culture

The U87 cell line was purchased from ATCC (cat. #HTB-14) and maintained in high-glucose DMEM (Gibco, cat. #11965) supplemented with 10% (v/v) fetal bovine serum (Gibco, cat. #10437028) and 1× antibiotic–antimycotic solution (Corning, cat. #30-004-Cl) and used up to passage 15. Primary NHAs were purchased from ScienCell (cat. #1800) and grown in astrocyte media (ScienCell, cat. #1801) and used up to passage 10 according to the manufacturer’s recommendations. Established and previously characterized PDX GBM cells (mesenchymal: GBM10 and GBM39; proneural: GBM85 and GBM117; and classical: GBM6 and GBM66) were obtained from Mayo Clinic Brain Tumor Patient-Derived Xenograft National Resource in serum-free media (23). PDX GBM cells were grown under serum-free tumor sphere–forming conditions: DMEM:F12 media (ATCC, cat. #30-2006) with 20 ng/mL human epidermal growth factor (Sigma-Aldrich, cat. #E9644), 20 ng/mL human basic fibroblast growth factor (Sigma-Aldrich, cat. #F0291), 1× antibiotic–antimycotic solution (Corning, cat. #30-004-Cl), and 1× vitamin B27 (Thermo Fisher Scientific, cat. #17504044) and used up to passage 10. All cell culture media was filter-sterilized (0.22 μm; Sigma-Aldrich, cat. #SCGP00525), and cells were maintained in a humidified incubator at 5% CO_2_ and 37°C. Short tandem repeat profiling of all cell lines was performed routinely to ensure authenticity.

### Cell number determination

Toxicity studies were determined by trypan blue dye exclusion. Adherent cells were detached and collected with trypsin–ethylenediaminetetraacetic acid (EDTA) (ATCC, cat. #30-2101); suspended tumor spheres were converted to single-cell suspensions with trypsin–EDTA and gentle pipetting with a 1,000-μL pipette tip. A 1:1 mixture of single-cell suspension and trypan blue dye was prepared, and live cells determined were with a hemacytometer using an inverted microscope (Olympus IMT-2).

For coculturing toxicity studies with U87 and NHAs, U87 cells were first fluorescently labeled with carboxyfluorescein succinimidyl ester (Invitrogen, cat. #C34554) per the manufacturer’s instructions. Labeled U87 cells were then seeded onto 12-well plates at a 1:1 ratio with unlabeled NHAs in DMEM. Cocultures were allowed to adhere and then treated with a titration of β-lap for 2 hours. After exposure, the media was replaced with fresh DMEM, and microscopic images were taken using an Echo microscope (bright-field and FITC channel 485/535 nm) 72 hours after exposure. Cell number and ratio were calculated using ImageJ (version 1.54, RRID: SCR_003070).

For clonogenic assays, U87 cells and NHAs were plated in 6-well plates with 500 and 1,000 cells per well, respectively. Cells were treated with β-lap with and without 10 nmol/L FK866 as in cytotoxicity studies. After exposure, plates were cultured 12 to 14 days with media replaced every 3 to 4 days. Colonies were fixed with 100% methanol for 15 minutes and stained with 0.5% crystal violet in 25% methanol. Colonies observed under microscopes containing more than 50 cells were counted.

### NAD^+^ measurement

The live cell number was determined using the trypan blue dye exclusion method. Cells were collected for NAD^+^ extraction, and NAD^+^ was normalized to the number of live cells. Intracellular NAD^+^ was measured using the enzyme-coupled reaction as described previously (24, 25) with modifications. The following stock solutions were prepared in deionized H_2_O: 1 mol/L Tris-HCl (pH 8.5), 1 mol/L Tris base (pH = 11), 0.1 mol/L HCl (pH = 1), 10 mg/mL phenazine methosulfate, 1 mg/mL MTT, and NAD^+^ standards (1–10 μmol/L). Fresh solution of 100 U/mL alcohol dehydrogenase was prepared in diH_2_O for each NAD^+^ measurement.

NAD^+^ extraction was performed by resuspending cell pellet in 50 μL of 0.1 mol/L HCl (pH = 1) and incubating at 60°C for 10 minutes. Solution was then neutralized by adding 50 μL of 0.4 mol/L Tris base (pH = 11) and centrifuged at 14,000 × *g* for 5 minutes. Twenty microliters of the supernatant or NAD^+^ standard (0–10 μmol/L) was then added to each well on a 96-well plate (Thermo Fisher Scientific, cat. #12565501) with 100 μL of the NAD^+^ reaction mixture (0.1 mol/L Tris-HCl, pH = 8.5; 0.9 mol/L ethanol; 2.7 mmol/L phenazine methosulfate; 0.7 mmol/L MTT; and 1 U alcohol dehydrogenase). Samples were incubated for up to 10 minutes at room temperature, and absorbance read at 600 nm (Eppendorf PlateReader AF2200). ΔOD_600_ was calculated by subtracting absorbance readings from blank. Levels of NAD^+^ per live cells were then calculated as follows:

$$\frac{\left[\text{NAD}+\right]\;µ\text{mol}/L}{10^{5}\;\text{live}\;\text{cells}}=\frac{\Delta {\text{OD}}_{600}}{\text{slope}\;\left[{\left(µ\text{mol}/L\right)}^{-1}\right]\times c\text{ell}\;\text{number}\;\left(\times 10^{5}\;\text{cells}\right)}$$

Data presented as % NAD^+^ of control.

### Western blot analysis

The protein concentration from cell lysates was determined using BCA assay. Samples were prepared in 1× Laemmli buffer to a final concentration of 1 μg/μL. Samples were denatured at 95°C for 5 minutes, and 20 μg protein from each sample was run on Mini-PROTEAN TGX gel. Proteins were transferred to membranes with the semidry method on the Trans-Blot Turbo transfer system (Bio-Rad, RRID: SCR_023156). Membranes were blocked with 5% milk in 1× TBS with 0.1% Tween-20 (TBS-T) for 1 hour at room temperature. Primary antibodies were diluted 1:1,000 in the blocking solution: NQO1 (Abcam, cat. #ab2346, RRID: AB_302995), nicotinamide phosphoribosyltransferase (NAMPT; Invitrogen, cat. #PA1-1045, RRID: AB_2104785), quinolinate phosphoribosyltransferase (QPRT) (Proteintech, cat. #25174-1-AP, RRID: AB_2879941), nicotinate phosphoribosyltransferase (NAPRT) (Abcam, cat. #ab211529, RRID: AB_3678660), and β-actin (Santa Cruz Biotechnology, cat. #sc47778, RRID: AB_626632). Membranes were washed 3 times with TBS-T before incubation with horseradish peroxidase (HRP)–conjugated secondary antibodies (1:2,000, Abcam): HRP-conjugated anti–goat IgG (cat. #ab205723, RRID: AB_3065024); HRP-conjugated anti–mouse IgG (cat. #ab205719, RRID: AB_2755049), and HRP-conjugated anti–rabbit IgG (cat. #ab205718, RRID: AB_2819160). Blots were imaged after exposing membranes to chemiluminescent substrate (Thermo Fisher Scientific, cat. #34577) using a ChemiDoc (Bio-Rad, RRID: SCR_019037) imaging system.

### Immunocytochemical staining

U87 cells (2 × 10^5^) were seeded onto cover glass slips in 35-mm cell culture dishes (Thermo Fisher Scientific, #153066) and allowed to adhere overnight. Cells were then exposed to vehicle (0.5% DMSO), β-lap (5 μmol/L), or β-lap (5 μmol/L) with dicoumarol (20 μmol/L) for 2 hours. Cells were then fixed in 5% paraformaldehyde, blocked with 5% BSA, and stained with anti-γH2AX antibody (Cell Signaling Technology, cat. #2577S, 1:400, RRID: AB_2118010) overnight at 4°C. The next day, primary antibody was washed with TBS-T, and samples were incubated with a secondary antibody (Cell Signaling Technology, cat. #8889, 1:1,000, RRID: AB_2716249) for 1 hour and mounted with antifade mounting solution [with 4′,6-diamidino-2-phenylindole (DAPI); Thermo Fisher Scientific, cat. #P36931] and visualized using an Echo microscope (RRID: SCR_026523). The mean fluorescence intensity of γH2AX staining was quantified and normalized to DAPI signal using ImageJ. For each sample, 3 fields of view were selected, and regions of interest corresponding to individual nuclei were manually outlined based on DAPI staining. The integrated density of γH2AX and DAPI signals was measured for each nuclei, and γH2AX intensity was normalized to that of DAPI. The normalized γH2AX/DAPI values were averaged per field and compared across treatment groups. All image acquisition settings were kept constant between conditions.

### Flow cytometry

For cell-cycle analysis, asynchronous U87 cells were treated with β-lap with and without 10 nmol/L FK866 as in cytotoxicity studies. After exposure, cells were trypsinized and collected in PBS and fixed in 70% ethanol. After fixation, cells were washed and resuspended in 0.1% Triton X-100 with 1 μg/mL DAPI. Cells were analyzed on CytoFLEX (Beckman Coulter, RRID: SCR_019627). At least 25,000 single-cell events were recorded per sample. Cell-cycle distribution was calculated on Floreada.io (https://floreada.io, RRID: SCR_025286).

### Caspase activity assay

The activation of apoptosis was evaluated with the fluorescent substrate for activated caspases 3 and 7: Ac-DEVD-AMC (Enzo Life, cat. #ALX-260-031) as previously described (26, 27). A total of 1 to 2 × 10^5^ cells were collected with trypsin–EDTA and pelleted (500 × *g* for 5 minutes). Cells were resuspended in 100 μL cold lysis buffer (50 mmol/L 4-(2-hydroxyethyl)-1-piperazineethanesulfonic acid, 1 mmol/L EDTA, and 0.1% Triton X-100) and left at 4°C for 15 to 30 minutes. The cell lysate was then centrifuged at 12,000 × *g* for 10 minutes at 4°C. Twenty microliters of the cell lysate was then added to 80 μL cell lysis buffer containing Ac-DEVD-AMC (final concentration per well of 20 μmol/L) and dithiothreitol (final concentration per well of 5 mmol/L). Samples were incubated at 37°C for 60 minutes. Fluorescence was measured at Excitation/Emission = 360/465 nm. For protein normalization, the protein concentration of each sample was measured using BCA assay. Caspase activity per μg protein was determined as previously described (26, 27). An arbitrary unit was defined as caspase activity normalized to control.

### Gene expression analysis

A combined dataset from The Cancer Genome Atlas (TCGA; http://cancergenome.nih.gov/) and noncancer patients from Genotype-Tissue Expression (GTEx) was accessed from the UCSC Xena platform TCGA TARGET GTEx study (https://xenabrowser.net, RRID: SCR_018938; ref. 28) as previously described (29). Due to the limited number of normal adjacent GBM tissue from TCGA, gene expression of GBM samples was compared with normal brain tissue of noncancer patients from GTEx. Log_2_-transformed gene expression data were obtained as “RSEM expected_count (DESeq2 standardized).” In total, 6,857 samples across 28 tissues (1,142 from brain tissue) from the GTEx database were used for gene expression comparison with 153 TCGA-GBM samples [140 isocitrate dehydrogenase (*IDH*) wild-type primary GBM tumors and 13 recurrent tumors).

### Statistics

All statistical and synergy analyses were performed on GraphPad Prism (version 10.0.0, RRID: SCR_002798) and CompuSyn (version 1.0, RRID: SCR_022931), respectively. IC_50_ values were determined using 4-parameter logistic regression. For cytotoxicity, DNA damage, NAD^+^ studies, and caspase activity, the appropriate statistical test was performed with *P* < 0.05 deemed statistically significant. Cell-cycle distribution for flow cytometry experiments were determined using the Watson Pragmatic model (30). Synergism was evaluated based on the Chou–Talalay method to generate combination indices (CI; ref. 31).

## Results

### NQO1 expression is elevated in U87 GBM cells and select PDX GBM cell lines and may be targeted by naphthoquinones

**Figure 1.**
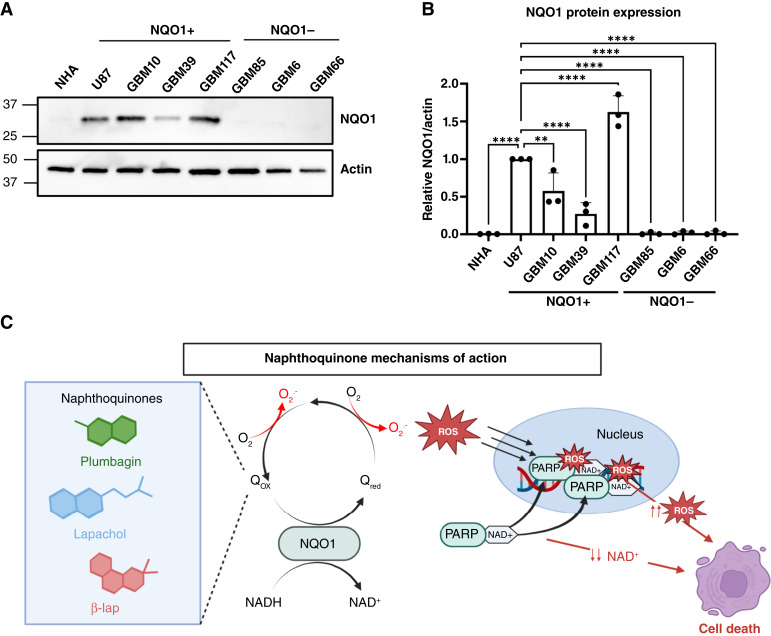
Targeting NQO1-expressing GBM with naphthoquinones. **A,** Western blot analysis demonstrating differential NQO1 expression across GBM cell lines. NQO1 expression was evaluated in NHAs, U87, and 6 PDX (GBM10, GBM39, GBM85, GBM117, GBM6, and GBM66) cell lines. Actin was used as the control. **B,** Quantification of Western blots. NQO1/actin expression was normalized to the reference U87 cell line. NQO1 expression is elevated in U87, GBM117, GBM10, and GBM39. **C,** Cartoon representation of reducible naphthoquinones including plumbagin, lapachol, and β-lap. Naphthoquinones may enter futile cycling by NQO1 reduction via NADH to generate reactive oxidative species (ROS). Accumulating ROS damage in DNA leads to PARP activation and NAD^+^ depletion. Severe ROS damage and NAD^+^ depletion lead to cell death. Q_ox_ and Q_red_ represent the oxidized and reduced states of naphthoquinone substrates, respectively. Values are mean ± SD of *n* = 3. ANOVA and Dunnett test; **, *P* < 0.01; ****, *P* < 0.0001. [**C,** Created in BioRender. Chang-Gu, B. (2026) https://BioRender.com/jhhxbyp.]

We investigated NQO1 protein expression in U87 GBM cells, 6 PDX GBM cell lines, and NHAs (Fig. 1A and B). NQO1 was highly expressed in U87 cells and 3 GBM cell lines (GBM10, GBM39, and GBM117) and was not expressed in GBM6, GBM66, GBM85, or NHAs (Fig. 1A and B). NQO1 may be therapeutically targeted by naphthoquinones including lapachol, plumbagin, and β-lap (17, 18). Naphthoquinones undergo futile cycling upon reduction by NQO1, leading to reactive oxidative species generation, NAD^+^ depletion, and ultimately cell death (Fig. 1C; refs. 11, 19).

### High NQO1-expressing GBM cells are sensitive to NQO1-activatable compounds

**Figure 2.**
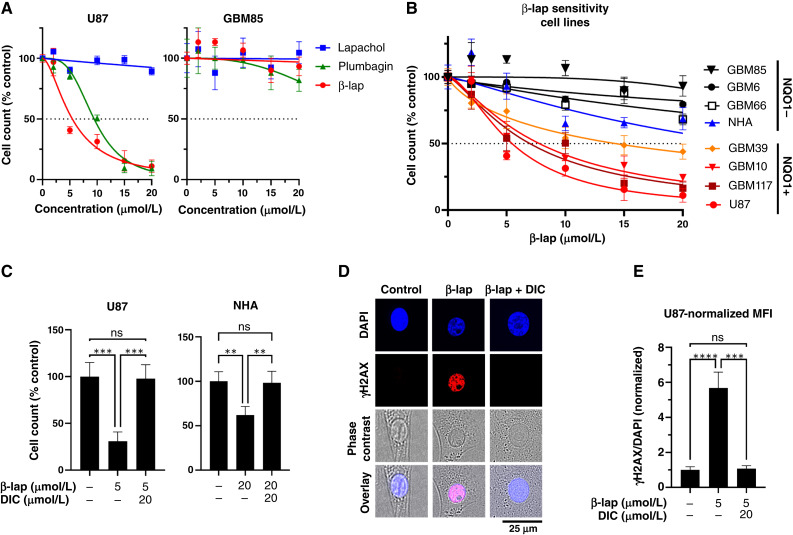
β-lap selectively targets NQO1-expressing GBM. **A,** Dose–response curves of naphthoquinone compounds in NQO1-expressing U87 and NQO1− GBM85 cells. U87 cells are sensitive to plumbagin and β-lap with IC_50_ values (95% confidence interval) of 9.5 (8.4–10.5) and 5.5 (4.2–6.9) μmol/L, respectively. U87 was largely insensitive to lapachol. None of the naphthoquinones exhibited significant toxicity in the NQO1− GBM85 cell line. **B,** Dose–response curve demonstrating higher sensitivity to β-lap in NQO1-expressing (NQO1+) cells. IC_50_ values of GBM10, GBM39, and GBM117 were 7.8 (6.1–9.8), 14.3 (9.9–27.3), and 7 (5.1–9.1) μmol/L, respectively. NHA, GBM6, and 66 were resistant to β-lap at concentrations up to 20 μmol/L. **C,** NQO1 inhibition with dicoumarol (DIC) reverses β-lap toxicity. U87 cells were exposed to 5 μmol/L β-lap for 2 hours with or without 20 μmol/L DIC, and cell viability was measured 24 hours after exposure. Similarly, NHA was exposed to 20 μmol/L β-lap for 2 hours with or without 20 μmol/L DIC. **D,** NQO1 inhibition reverses β-lap–induced DNA damage. U87 cells were exposed to 5 μmol/L β-lap with and without 20 μmol/L DIC for 2 hours. Cells were stained for γH2AX (red) and counterstained with DAPI (blue) for nucleus, with representative images. **E,** γH2AX/DAPI-normalized fluorescence intensity. The mean fluorescence intensity (MFI) in **D** was quantified and presented as the ratio of γH2AX/DAPI normalized to untreated control. Values are mean ± SD of *n* ≥ 3. ANOVA and Tukey multiple comparison test; **, *P* < 0.01; ***, *P* < 0.001; ****, *P* < 0.0001; ns, not statistically significant.

To evaluate the potential of NQO1 being a selective target in GBM, we identified multiple naphthoquinones (plumbagin, β-lap, and lapachol). Functional studies were performed in U87 cells as the cell line is widely used in GBM research and commercially available to most labs. ARQ761, a derivative of β-lap, demonstrated modest single-agent activity against refractory NQO1-expressing solid tumors in phase I clinical trials with a peak serum concentration of 8 μmol/L and a central compartment half-life of 2.6 hours (32). Thus, we exposed both the NQO1-expressing U87 cell line and the NQO1− GBM85 cell line to naphthoquinones for 2 hours and evaluated the cell number 24 hours after exposure. U87 cells were sensitive to β-lap and plumbagin with calculated IC_50_ values (95% confidence interval) of 5.5 (4.2–6.9) and 9.5 (8.4–10.5) μmol/L, respectively. Lapachol was not found to be cytotoxic to U87 cells within our exposure time (Fig. 2A). Additionally, both β-lap and plumbagin induced concentration-dependent depletion in NAD^+^ levels in U87 cells after initial 2-hour exposure but not lapachol (Supplementary Fig. S1). None of the compounds induced significant toxicity in the NQO1− GBM85 cell line (Fig. 2A). As β-lap was the most toxic naphthoquinone to U87 cells while sparing the NQO1− GBM85 cell line, we next evaluated β-lap toxicity in the remainder of our PDX GBM cell lines. Among PDX GBM, high NQO1-expressing cell lines, GBM10, GBM39, and GBM117, were sensitive to β-lap treatment with IC_50_ values of 7.8 (6.1–9.8), 14.3 (9.9–27.3), and 7 (5.1–9.1) μmol/L, respectively (Fig. 2B). PDX GBM with low NQO1 expression (GBM6, 66, and 85) and NHA remained resistant to β-lap (Fig. 2B). To further demonstrate β-lap selectivity of NQO1-expressing GBM, NHAs were cocultured with fluorescently labeled U87 in DMEM. Seventy-two hours after treatment, β-lap selectively targeted U87 cells at concentrations above 10 μmol/L (Supplementary Fig. S2). At high concentrations, β-lap induced some toxicity in NHAs (cell count of 70% of the control; Fig. 2B). To demonstrate that the toxic effects of β-lap are NQO1 dependent, we performed studies with the NQO1 inhibitor dicoumarol in both U87 cells and NHAs (Fig. 2C). Dicoumarol reversed β-lap toxicity in U87 cells treated with 5 μmol/L β-lap and NHA treated with 20 μmol/L β-lap. To evaluate β-lap–induced DNA damage, we performed γH2AX staining and observed significant DNA damage within 2 hours of exposure in U87 cells, and this damage was reversed upon the addition of dicoumarol (Fig. 2D and E).

### NQO1-expressing GBM may recover from β-lap–induced NAD^+^ depletion by resynthesizing NAD^+^

**Figure 3.**
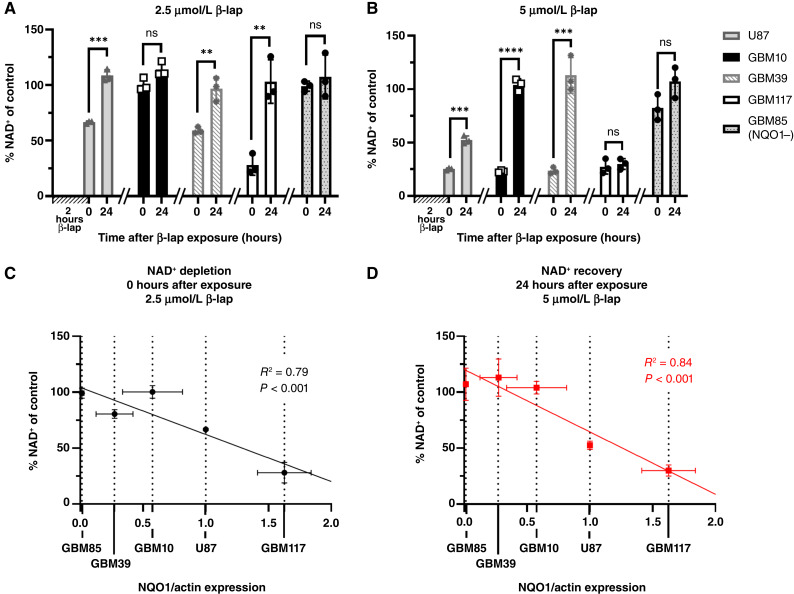
NQO1-expressing cells may recover from β-lap–induced NAD^+^ depletion. GBM10, GBM39, GBM117, and U87 were exposed to vehicle control (0.1% DMSO) or β-lap (2.5 and 5 μmol/L) for 2 hours, and NAD^+^ was evaluated 0 and 24 hours after exposure. The NQO1− GBM85 cell line was used as the negative control. **A** and **B,** β-lap induces acute NAD^+^ depletion. β-lap induces NAD^+^ depletion across NQO1-expressing cell lines with the most depletion observed at 5 μmol/L β-lap. **C,** NAD^+^ depletion is correlated with NQO1 expression. Lower NQO1 expression was associated with less NAD^+^ depletion 0 hours after 2.5 μmol/L β-lap exposure. **D,** NAD^+^ recovery is correlated with NQO1 expression. Higher NQO1 expression was associated with less NAD^+^ regeneration 24 hours after 5 μmol/L β-lap exposure. Values are mean ± SD of *n* = 3. *t* test (**A** and **B**), linear regression with goodness of fit (R^2^), and statistical significance shown on graph (**C** and **D**). **, *P* < 0.01; ***, *P* < 0.001; ****, *P* < 0.0001; ns, not statistically significant.

The primary mechanism for β-lap toxicity has been reported to be through NAD^+^ depletion (16). To evaluate β-lap’s effects on NAD^+^, we treated NQO1-expressing GBM cells with 2.5 and 5 μmol/L β-lap for 2 hours as these concentrations were close to the IC_50_ across cell lines. We subsequently evaluated NAD^+^ levels in live cells 0 and 24 hours after β-lap exposure. NAD^+^ measurements were normalized based on the number of live cells to ensure that cytotoxicity did not skew results. β-lap induced an acute concentration-dependent NAD^+^ depletion in high NQO1-expressing cells but not in the NQO1− GBM85 cell line (Fig. 3A and B). Despite significant initial NAD^+^ depletion, there was minimal initial toxicity (Fig. 3A and B; Supplementary Fig. S3). Cell death was observed only 24 hours after initial exposure, demonstrating that NAD^+^ depletion preceded cell death (Supplementary Fig. S3). We observed NQO1 expression to be correlated with NAD^+^ depletion across the 5 cell lines we evaluated (Fig. 3C). However, in all NQO1-expressing GBM cells, cells can recover from acute NAD^+^ depletion within 24 hours of initial β-lap exposure. We observed the highest rates of NAD^+^ recovery in lower NQO1-expressing cells (GBM10 and GBM39; Fig. 3D). The NQO1− GBM85 cell line did not experience significant changes in NAD^+^ levels after β-lap exposure (Fig. 3).

### The NAD^+^ salvage pathway is present in all GBM cells, and the Preiss–Handler pathway rescues NAD^+^ only in NAPRT-expressing cells

**Figure 4.**
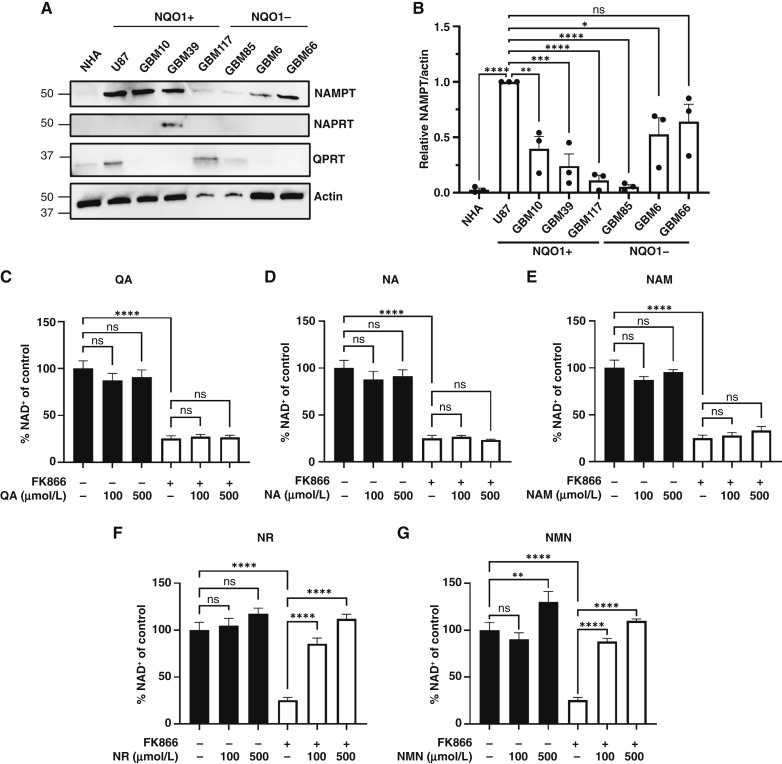
Salvage pathway is the primary NAD^+^ synthesis pathway active in NQO1-expressing GBM. **A,** Western blots of phosphoribosyltransferases expression across cell lines. Expression of proteins involved in the salvage (NAMPT), *de novo* (QPRT), and Preiss–Handler (NAPRT) NAD^+^ biosynthesis pathways was evaluated. **B,** Quantification of NAMPT expression in cells. **C–E,** QA, NA, and NAM supplementation did not rescue NAD^+^ upon NAMPT inhibition with FK866 in U87 cells. FK866 (10 nmol/L) induces significant NAD^+^ depletion. QA, NA, and NAM (100 and 500 μmol/L) did not increase NAD^+^ levels in untreated (black bars) or in FK866-treated cells (white bars). **F** and **G,** NR and NMN addition rescued NAD^+^ upon NAMPT inhibition with FK866 in U87 cells. NR and NMN (100 and 500 μmol/L) increased NAD^+^ levels in FK866-treated cells (white bars) in a concentration-dependent manner. Values are mean ± SD of *n* = 3; ANOVA and Dunnett multiple comparison (**B**) or Tukey multiple comparison test (**C–G**); *, *P* < 0.05; **, *P* < 0.01; ***, *P* < 0.001; ****, *P* < 0.0001; ns, not statistically significant.

Regeneration of NAD^+^ after β-lap exposure potentially allows GBM to evade β-lap toxicity. To evaluate the active NAD^+^ pathways in GBM, we first evaluated protein expression levels of phosphoribosyltransferases involved in NAD^+^ biosynthesis across cell lines (Fig. 4A). QPRT, NAPRT, and NAMPT couple quinolinic acid (QA), nicotinic acid (NA), and nicotinamide (NAM), respectively, to phosphoribosyl pyrophosphate. NAMPT facilitates the NAD^+^ salvage pathway and is expressed in all GBM cells but not NHAs. The highest NAMPT expression was observed in U87 followed by GBM66, GBM6, GBM10, GBM39, GBM117, and GBM85 (Fig. 4A and B). QPRT facilitates *de novo* NAD^+^ synthesis, and its expression was detectable in NHAs, U87, and GBM117 (Fig. 4A). NAPRT facilitates the Preiss–Handler pathway for NAD^+^ synthesis, and its expression was only found in GBM39 (Fig. 4A). We then performed NAD^+^ precursor studies in all NQO1-expressing GBM cell lines (U87, GBM10, GBM39, and GBM117; Fig. 4C–G). In all tested GBM cells, we found that exposure to the NAMPT inhibitor FK866 (10 nmol/L) for 24 hours induces NAD+ depletion (Fig. 4; Supplementary Fig. S4). We show that supplementation with NAM had no effects on NAD^+^ in FK866-treated cells and supplementation with nicotinamide riboside (NR) and nicotinamide mononucleotide (NMN) rescued NAD^+^ depletion in all FK866-treated GBM cells (Fig. 4; Supplementary Fig. S4). Surprisingly, supplementation with the pyridine QA did not rescue NAD^+^ in any of the tested GBM cell lines regardless of QPRT expression status (Fig. 4; Supplementary Fig. S4). Finally, supplementation with NA rescued NAD^+^ only in the NAPRT-expressing GBM39 cell line (Fig. 4; Supplementary Fig. S4).

### Inhibition of the NAD^+^ salvage pathway increases β-lap toxicity by inhibiting NAD^+^ recovery after β-lap exposure

**Figure 5.**
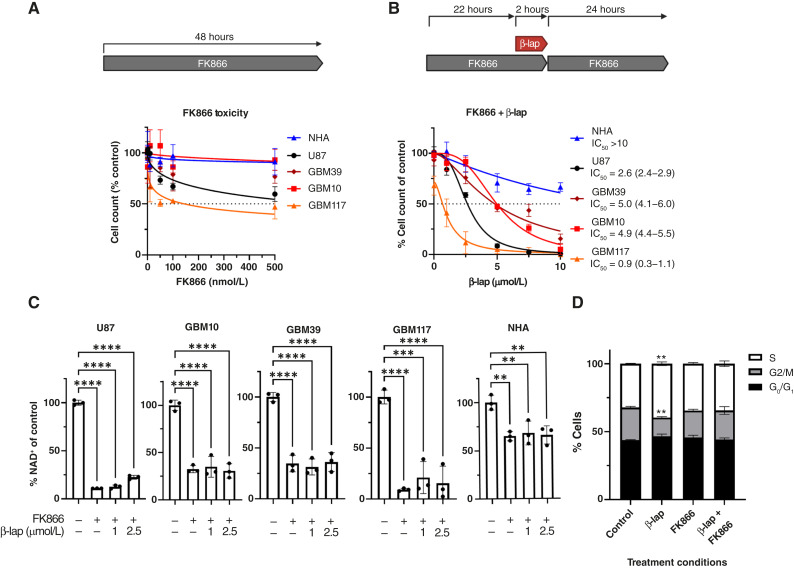
Combination treatment with FK866 and β-lap blocks NAD^+^ regeneration and increases β-lap cytotoxicity. **A,** FK866 has limited single-agent toxicity. Dose–response curves of cells exposed to FK866 (0–500 nmol/L) for 48 hours. **B,** FK866 increases β-lap toxicity in NQO1-expressing GBM. Dose–response curves of cells exposed to 10 nmol/L FK866 and β-lap (0–10 μmol/L). IC_50_ values of U87, GBM39, GBM10, and GBM117 were 2.6 (2.4–2.9), 5 (4.1–6), 4.9 (4.4–5.5), and 1 (0.5–1.5) μmol/L, respectively. NHAs remained resistant to β-lap. **C,** FK866 blocks NAD^+^ regeneration in NQO1-expressing GBM. Cells were exposed to 10 nmol/L FK866 and β-lap (1 or 2.5 μmol/L) in the same manner as in **B**, and NAD^+^ was quantified 24 hours after β-lap exposure. **D,** Cell-cycle analysis of U87 cells. Cells were exposed to FK866 (10 nmol/L) and/or β-lap (5 μmol/L) in the same manner as in **B**. Cells were fixated and analyzed 24 hours after β-lap exposure. Values are mean ± SD of *n* ≥ 3; ANOVA and Tukey multiple comparison test; **, *P* < 0.01; ***, *P* < 0.001; ****, *P* < 0.0001. [Top graphics in **A** and **B** were created in BioRender. Chang-Gu, B. (2026) https://BioRender.com/jhhxbyp.]

As the NAD^+^ salvage pathway was active in all tested NQO1-expressing GBM cells, we next evaluated the effects of NAMPT inhibition on β-lap toxicity. Clinical trials with FK866 have demonstrated the maximum steady-state serum concentration of FK866 to be 14 nmol/L that was maintained for over 80 hours (33). We first exposed NQO1-expressing GBM cells (U87, GBM10, GBM39, and GBM117) to FK866 for 48 hours and demonstrated that it has limited single-agent toxicity up to 500 nmol/L in all cells except GBM117 [IC_50_ = 60.3 (35.8–106.2) nmol/L; Fig. 5A]. Previous studies demonstrated that pretreatment with FK866 primed pancreatic cancer cells to β-lap by lowering NAD^+^ pools within cells (16). We found that U87 cells required continuous FK866 exposure before, during, and after β-lap treatment to increase β-lap toxicity (Supplementary Fig. S5; Fig. 5B). Thus, this continuous exposure was used for all subsequent GBM experiments (Fig. 5B). We found that the addition of FK866 significantly decreased the IC_50_ of β-lap in NQO1-expressing GBM cells (Fig. 5B). For comparison, we performed the same studies in NHAs. There was no toxicity of FK866 observed against NHAs, and NHAs remained resistant to the combination of β-lap and FK866 (Fig. 5A and B). Additional clonogenic assays in U87 cells and NHAs demonstrate similar effects with U87 cells more sensitive to β-lap and the combination of β-lap and FK866 than NHAs (Supplementary Fig. S6). U87 exhibited slightly greater sensitivity to β-lap by clonogenic assay compared with cell counting assay, consistent with clonogenic assay’s ability to capture delayed cell death and loss of proliferative capacity. Using the Chou–Talalay method, we show that the combination of FK866 and β-lap exhibits significant synergy against NQO1-expressing GBM (Table 1). Of interest, the combination was most toxic toward the GBM117 cell line. Additionally, despite NHAs remaining resistant to the combination of FK866 and β-lap, the combination was still synergistic with a CI of 0.38 ± 0.13. We found that FK866 blocks NAD^+^ regeneration after β-lap exposure and increases β-lap toxicity in NQO1-expressing GBM cells while still sparing the non–NQO1-expressing NHAs (Fig. 5C). Additional cell-cycling experiments were performed in U87 cells. β-lap (5 μmol/L) treatment induced an increase in the S-phase population and decrease in the G_2_–M population. However, FK866 (10 nmol/L) and the combination of β-lap with FK866 led to no changes in cell-cycle distribution relative to control (Fig. 5D; Supplementary Fig. S7). Additional toxicity studies demonstrated that the combination of β-lap (5 μmol/L) and FK866 (10 nmol/L) induced rapid cell death within 4 hours after β-lap exposure (Supplementary Fig. S8). We then evaluated the activation of apoptotic pathways with the caspase 3/7 fluorogenic substrate AC-DEVD-AMC. β-lap exposure led to eventual caspase 3/7 activation, consistent with the activation of apoptotic cell death pathways. However, neither combination of FK866 and β-lap nor 10 nmol/L FK866 alone induced caspase activation (Supplementary Fig. S7). These results suggest that NAMPT inhibition sensitizes NQO1-expressing GBM to β-lap and induces a rapid caspase 3/7–independent cell death.

**Table 1.** Combination treatment of FK866 and β-lap is synergistic in NQO1-expressing GBM.

Cell line	FK866 (nmol/L)	β-lap (μmol/L)	10 nmol/L FK866 + β-lap (μmol/L)	CI ± SD
U87	>500	5.5 (4.2–6.9)	2.6 (2.4–2.9)	0.25 ± 0.06
GBM10	>500	7.8 (6.1–9.8)	4.9 (4.4–5.5)	0.46 ± 0.03
GBM39	>500	14.3 (9.9–27.3)	5 (4.1–6)	0.29 ± 0.11
GBM117	60.3 (35.8–106.2)	7 (5.1–9.1)	1 (0.5–1.5)	0.11 ± 0.08
NHAs	>500	>20	>10	0.38 ± 0.13

NOTE: IC_50_ values with 95% confidence intervals were determined based on 48-hour FK866 exposure, 2-hour β-lap exposure, or 2-hour β-lap exposure with FK866 for 48 hours. CIs with SD were calculated using CompuSyn at 5 μmol/L β-lap and 10 nmol/L FK866. CI < 1 indicates synergism; CI = 1 indicates additive; and CI > 1 indicates antagonism.

Abbreviation: SD, standard deviation.

### NQO1 expression is epigenetically controlled

**Figure 6.**
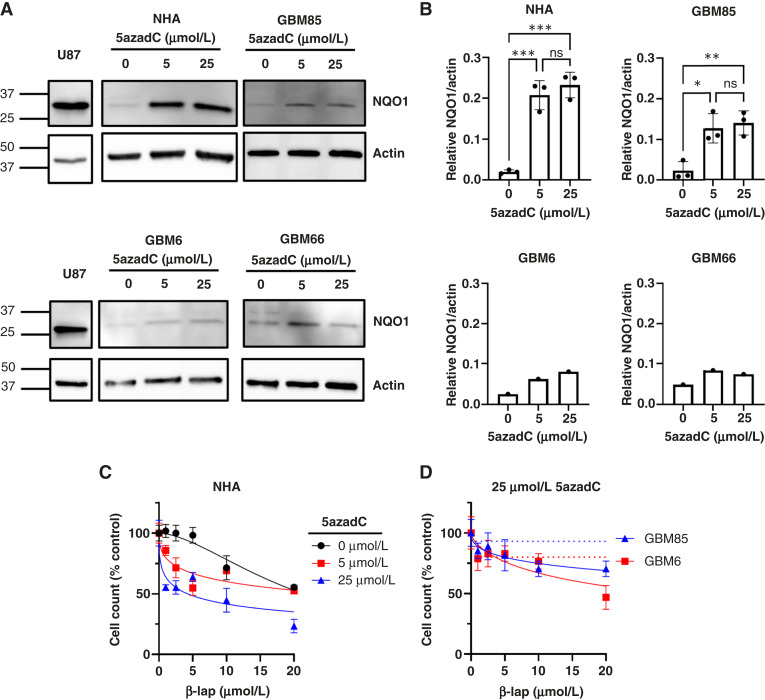
NQO1 expression is induced in low NQO1-expressing cells with 5azadC. Cells were exposed to 0, 5, and 25 μmol/L 5azadC for the cell-doubling times. **A,** NQO1 protein expression via Western blot analysis. NQO1 expression was elevated upon exposure to 5azadC in NHAs (3 days), GBM85 (5 days), GBM6 (8 days), and GBM66 (10 days). **B,** Quantified NQO1/actin expression. Ratio of NQO1/actin expression was normalized to U87. Values for NHAs and GBM85 are mean ± SD of *n* = 3; ANOVA and Tukey multiple comparison test; *, *P* < 0.05; **, *P* < 0.01; ***, *P* < 0.001; ns, not statistically significant. Values for GBM6 and GBM66 are *n* = 1. **C,** β-lap kill curves of 5azadC-exposed NHAs. **D,** β-lap kill curves of 5azadC-exposed GBM85 and GBM6. Dashed line represents previously observed cell count after exposure to 20 μmol/L β-lap in unexposed GBM85 (blue) and GBM6 (red) cells (Fig. 2). Values are mean ± SD of *n* = 3.

It has previously been reported that NQO1 expression in human liver cancer cells is regulated by methylation of the NQO1 promoter (34, 35). We further investigated if demethylation may increase NQO1 expression in our non–NQO1-expressing cell lines. To test this, we exposed our low NQO1-expressing PDX GBM cell lines (GBM6, GBM66, and GBM85) as well as NHAs to the demethylating agent 5-Aza-2′-deoxycytidine (5azadC) for one cell-doubling period. We found that 5azadC exposure increased NQO1 protein expression levels in NHAs and GBM85 cells (Fig. 6A and B). After 25 μmol/L 5azadC exposure, relative NQO1 expression levels in NHAs and GBM85 cells were 30% and 15%, respectively, of U87 (Fig. 6B). A modest increase in NQO1 was also observed after 5azadC exposure in the slower proliferating GBM6 and GBM66 cell lines (Fig. 6A and B). We then treated 5azadC-exposed NHAs as well as GBM85 and GBM6 cells to β-lap. After 5azadC treatment, these cells became more sensitive to β-lap (Fig. 6C and D).

### NQO1 and NAMPT expression in GBM and human tissue

**Figure 7.**
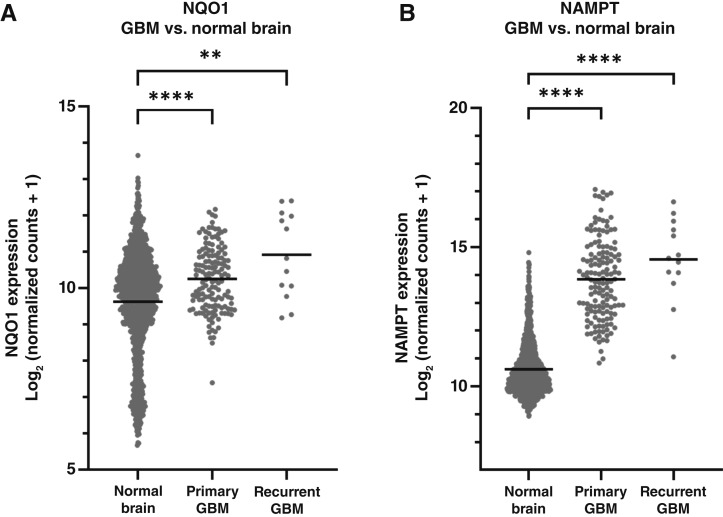
NQO1 and NAMPT expression is elevated in GBM. Gene expression data from TCGA-GBM and GTEx were accessed through UCSC Xena Browser. Normal brain tissue from noncancer patients (GTEx, *n* = 1,142) were compared with primary IDH wild-type GBM tumors (*n* = 140) and recurrent GBM tumors (*n* = 13). **A,** NQO1 expression in brain tissue. **B,** NAMPT expression in brain tissue. Statistical significance was determined using ANOVA with the Dunnett multiple comparison test. **, *P* < 0.01; ****, *P* < 0.0001.

Gene expression analysis of GBM tissue from TCGA and normal tissue from the GTEx project demonstrates NQO1 and NAMPT expression to be elevated in both primary and recurrent GBM compared with normal brain tissue (Fig. 7A and B). Expression levels of NQO1 and NAMPT across human tissue were then evaluated (Supplementary Fig. S9). NQO1 expression was found to be highly expressed in normal stomach tissue, adrenal glands, and adipose tissue. NAMPT was highly expressed in whole blood, liver, and lung tissue.

## Discussion

GBM is a highly heterogeneous tumor, often comprising multiple cellular subpopulations. Early gene expression data classified GBM into at least 3 distinct subtypes: mesenchymal, proneural, and classical (5, 6, 36, 37). These phenotypes may vary within the tumor and change as a function of time because of intratumoral heterogeneity and GBM plasticity (38). Plasticity of GBM is of particular interest as proneural-to-mesenchymal transition is thought to drive recurrence and therapy resistance (38–40). Although prior studies have expanded our understanding of GBM subtypes and identified potential subtype-specific therapeutic targets, no FDA-approved treatments currently target GBM based on these molecular phenotypes (8, 39, 41, 42). In contrast to subtype-specific approaches, gene expression data may directly identify vulnerabilities in GBM. Here, we used a panel of previously characterized PDX GBM lines from Mayo Clinic to better understand GBM susceptibility to NQO1-targeting treatments.

NQO1 is a cytoprotective oxidoreductase that detoxifies quinones through 2-electron reduction and is transcriptionally activated via the nuclear factor erythroid 2–related factor 2 (Nrf2) in response to oxidative stress (43, 44). Previously, Pölönen and colleagues (45) had identified Nrf2 to be hyperactivated in mesenchymal GBM and linked to GBM progression. NQO1 is a well-understood target for Nrf2 transcription activation, and in 2023 a different group reported NQO1 expression to be upregulated in mesenchymal GBM and NQO1 to drive mesenchymal transition in GBM (15). These previous reports suggest that NQO1 expression correlates with GBM molecular subtypes, and thus we used a panel of previously characterized PDX GBM cell lines from Mayo Clinic (23) for our studies and demonstrate that multiple GBM cell lines and subtypes including U87, GBM10 (mesenchymal), GBM39 (mesenchymal), and GBM117 (proneural) expressed high levels of NQO1 (Fig. 1). Previously, it has been shown that cancer cells expressing NQO1 can selectively bioactivate cytotoxic agents, targeting cancers including non–small cell lung cancer, pancreatic cancer, and breast cancer (11, 16, 46, 47). Here, we demonstrate that NQO1 expression in GBM, regardless of GBM subtype, determines sensitivity to the NQO1-targeting compound β-lap.

Among the naphthoquinones tested (plumbagin, lapachol, and β-lap), we report that β-lap was the most potent against U87 GBM cells (Fig. 2). We demonstrate that NQO1-expressing GBM was sensitive to β-lap, whereas GBM85, GBM6, GBM66, and NHAs had little NQO1 expression and were resistant (Fig. 2). The IC_50_ for sensitive cells was approximately 5 to 15 μmol/L, and across GBM cell lines cell lines with higher NQO1 expression (Fig. 1B) were more sensitive to β-lap (U87 and GBM117). Coculture experiments further confirmed that U87 cells were uniquely sensitive to β-lap treatment compared with NHAs (Supplementary Fig. S2). NQO1 inhibitor, dicoumarol, reversed both the toxicity and acute DNA damage induced by β-lap, confirming the NQO1 dependence of β-lap’s mechanism (Fig. 2). Notably, dicoumarol also reversed the modest toxicity observed in NHAs treated with high β-lap concentrations, suggesting that residual β-lap toxicity in NHAs is NQO1 dependent. Previously ARQ761, a β-lap analogue, had modest effects in phase I clinical trials toward improving outcomes of advanced non-CNS NQO1+ solid tumors (32). The dose-limiting toxicity (DLT) for patients was anemia, presumed to be due to methemoglobinemia (32). However, less is known about NQO1 as a target for CNS tumors. To further evaluate the selectivity of NQO1- and NAMPT-targeting compounds in GBM, we evaluated gene expression levels of NQO1 in TCGA-GBM samples as well as normal tissues from the GTEx database (Fig. 7). Both NQO1 and NAMPT were found to be elevated in primary and recurrent GBM compared with normal brain tissue. Across human tissues, NQO1 was elevated in normal gastrointestinal (GI) tissue, suggesting that NQO1-targeting compounds may exhibit significant off-target effects in the GI tract. Systemic side effects of NQO1-targeting compounds may be limited with the addition dicoumarol, a compound which belongs to a family of warfarin compounds and a potent inhibitor of NQO1. Dicoumarol does not cross the blood–brain barrier (48) and may be a particularly effective compound to be given in combination with β-lap to limit unwanted peripheral toxicity of β-lap. However, further work will be required to determine if combination of dicoumarol and β-lap is an effective strategy in targeting CNS malignancies while sparing peripheral cells.

A key finding of this study is that β-lap–induced NAD^+^ depletion in GBM is transient. When β-lap and other naphthoquinones are activated by NQO1, redox cycling generates superoxide, which damages genomic DNA. Damaged DNA activates DNA repair pathways, including PARP, which in turn depletes NAD^+^ levels (11, 19, 20, 49, 50). NAD^+^ depletion has previously been reported as a crucial mechanism for NQO1-targeting drugs’ toxicity (16, 20). Here, we show that β-lap induces acute DNA damage and NAD^+^ depletion in NQO1-expressing cells (Figs. 2 and 3). However, we further observe that NAD^+^ levels in surviving GBM cells may be recovered within 24 hours, suggesting that NAD^+^ regeneration allows GBM to evade β-lap toxicity (Fig. 3).

We next evaluated which pathway is primarily responsible for NAD^+^ regeneration in β-lap–treated GBM (Fig. 4). Within the tumor microenvironment, stressors such as hypoxia and ischemia trigger the release of cellular contents, resulting in elevated local concentrations of nucleotides and their precursors (51, 52). Under extreme metabolic stress, cancer cells can scavenge these extracellular nucleotides to support survival (53). Salvage of extracellular nucleotide precursors may allow cancer cells to restore NAD^+^ in response to NAD^+^-depleting agents including β-lap and evade cell death. There are 3 pathways for NAD^+^ synthesis that utilize different pyridine sources: the *de novo* (QPRT), Preiss–Handler (NAPRT), and salvage (NAMPT) pathways. Multiple pathways for NAD^+^ synthesis have been reported to be active in GBM (54, 55); however, the primary pathway for NAD^+^ synthesis in GBM is thought to be via the salvage pathway in which NAM is coupled to phosphoribosyl pyrophosphate by NAMPT (9, 55).

We performed immunoblotting and observed NAMPT expression in all GBM cells but not in NHAs. QPRT was expressed in U87 and GBM117 cells, and NAPRT was only expressed in GBM39. We evaluated the activity of each pathway by performing NAD^+^ precursor studies. We found that NAMPT inhibition with FK866 led to NAD^+^ depletion in all tested GBM cell lines (Fig. 4; Supplementary Fig. S4). Supplementation with pyridine nucleosides downstream of NAMPT (NR and NMN) rescued NAD^+^ in all FK866-treated cells (Fig. 4; Supplementary Fig. S4). These pyridine nucleosides contain sugars and do not require activity of phosphoribosyltransferases for NAD^+^ synthesis and have previously been demonstrated to rescue NAD^+^ in NAMPT-inhibited cells (55, 56). The Preiss–Handler pathway was found to only be active in 1 GBM cell line expressing NAPRT (GBM39). This NAPRT-dependent activity of the Preiss–Handler pathway in GBM was similarly observed in a recent study (55). Surprisingly, we found that regardless of QPRT expression, QA did not rescue NAD^+^ levels in FK866-treated GBM cells. Although previous studies have demonstrated QA to increase GBM cell viability upon exposure to FK866, none have demonstrated QA to increase intracellular NAD^+^ levels (54, 55). We reason that although the *de novo* pathway may be active in GBM, this pathway is likely not responsible for the observed NAD^+^ regeneration after acute NAD^+^ depletion. Taking our immunoblot and NAD+ precursor studies together, we demonstrate the NAD^+^ salvage pathway is active in all GBM cell lines, and we next evaluated if NAMPT inhibition with FK866 may increase β-lap toxicity.

We first investigated single-agent toxicity of FK866 toward NQO1-expressing GBM cells and NHAs. Although all tested cells experienced NAD^+^ depletion with FK866 exposure, only GBM117, the tested GBM cell line with the lowest NAMPT expression, was sensitive to single-agent FK866 treatment (Figs. 4 and 5). We next evaluated the combination of FK866 and β-lap on GBM toxicity. Previous studies in pancreatic cancer and NSCLC have suggested that FK866 primes cells to β-lap by lowering baseline NAD^+^ levels before β-lap exposure (16, 21, 22). However, our findings suggest that FK866 enhances β-lap toxicity in GBM by preventing NAD^+^ regeneration following β-lap–induced depletion. As priming alone failed to change β-lap toxicity in GBM, this suggests that GBM possesses a high capacity to regenerate NAD^+^ following initial β-lap insult. Using the Chou–Talalay method, we demonstrate this combination strategy to be strongly synergistic in NQO1-expressing GBM cells with CIs between 0.11 and 0.46 (Table 1; ref. 31). Chou–Talalay analysis also indicated a synergistic CI in NHAs (0.38); however, the absolute reduction in NHA viability after exposure remained modest compared with the tested NQO1+ GBM cell lines (Table 1). This is likely due to the low NQO1 expression and β-lap activation in NHAs, such that the absolute effects of the combination treatment are limited in NHAs. Of the tested cells, GBM117 was found to be particularly sensitive to the combination of FK866 and β-lap. Although FK866 failed as monotherapy in clinical trials due to DLT and modest single-agent efficacy, our data demonstrate that physiologically achievable concentrations of both FK866 (10 nmol/L) and β-lap (<10 μmol/L) produce significant cytotoxicity against NQO1-expressing GBM while sparing NHAs (Fig. 5; refs. 32, 33).

We observe that β-lap induces an S-phase delay in U87 GBM cells similar to previous reports in breast, prostate, and colon cancer cell lines (Fig. 5D; refs. 57–59). This delay has previously been reported to be due to β-lap induction of E2F1, a transcription factor that regulates DNA damage repair and S-phase progression in cells (58, 60). Under low oxidative stress and temporary NAD^+^ depletion, cells may replenish intracellular energy stores and preferentially activate apoptotic pathways (27). Consistent with this model, we observed that β-lap induced an S-phase delay and activation of apoptosis likely due to DNA damage response following a partial recovery of acute NAD^+^ depletion (Fig. 5D; Supplementary Fig. S8; ref. 27). However, the addition of FK866 with β-lap prevented NAD^+^ regeneration and induced a rapid cell death without activation of caspase 3/7 (Supplementary Fig. S7). Excessive NAD^+^ depletion due to PARP activity following initial DNA damage may cause cell death that is distinct from apoptosis (16, 61). Thus, our results suggest the mechanism of β-lap toxicity, such as general oxidative stress, is context dependent (27). Under low oxidative stress, GBM cells may regenerate NAD^+^ and induce activation of apoptotic pathways or repair DNA damage. However, under excessive NAD^+^ depletion, by either higher β-lap concentration or NAMPT inhibition with FK866, cells undergo a bioenergetic collapse and rapid cell death.

NQO1 expression has been reported to be epigenetically controlled by methylation of the NQO1 promoter (34, 35). Previously, Tada and colleagues (34) reported that exposure to the demethylating agent 5azadC significantly decreased methylation within the NQO1 promoter region and increased NQO1 RNA expression in liver cancer cells. Here, we report that 5azadC exposure increased NQO1 expression in NHAs as well as in our low NQO1-expressing PDX GBM lines (Fig. 6). Relative NQO1 expression in NHAs and GBM85 increased to 15% to 30% of U87 levels and became more sensitive to β-lap, consistent with NQO1’s role in β-lap toxicity. We observed a modest increase in NQO1 expression in 2 of our PDX GBM cell lines (GBM6 and GBM66), both of which exhibited slower doubling times. This limited response may be either due to reduced incorporation of 5azadC during replication or alternative regulatory mechanisms for NQO1 expression. Interestingly, previous global methylation profiling studies have classified our PDX GBM cell lines to be hypomethylated relative to *IDH*-mutant low-grade gliomas (23, 62). *IDH*-mutant gliomas exhibit global hypermethylation because of accumulation of the demethylase inhibitor 2-hydroxyglutarate (63). Despite the global hypomethylation reported within GBM, we suspect that differential methylation patterns of specific promoters of interest may still regulate NQO1 expression. Further work will be required to understand the primary regulatory mechanisms for NQO1 expression status in GBM.

This study has several limitations. First, our study is limited because of the lack of *in vivo* studies to further validate therapeutic efficacy, systemic toxicity, and pharmacokinetics (PK). Previous *in vitro* reports have demonstrated β-lap and other naphthoquinones to be blood–brain barrier permeable, and a recent *in vivo* study published while this article was under review has demonstrated the efficacy of intraperitoneally injected β-lap in an orthotopic xenograft glioma model (19, 64, 65). Future studies will be required to determine the PK profile of these compounds and the combination with FK866. Second, our gene expression studies utilize bulk transcriptomic data from TCGA and GTEx, and we are unable to report on NQO1 expression at the cellular level. Recent single-cell RNA sequencing (RNA-seq) studies have demonstrated NQO1 to be highly expressed in GBM-associated glioma stem cells compared with neural stem cells (65); however, future single-cell RNA-seq approaches may be useful for evaluation of NQO1 and NAMPT expression across cell types. Third, β-lap may interact with other redox-active enzymes, and the combination with NAMPT inhibition could amplify metabolic stress in unintended cell populations. *In vivo* studies establishing the therapeutic index of this combination will be required before they may be evaluated as therapeutic candidates. Despite these limitations, our study provides a thorough mechanistic understanding of NQO1-targeted therapy effects on intracellular NAD^+^ levels across multiple patient-derived GBM cell lines, and future *in vivo* studies are required to determine the clinical feasibility of NQO1-targeted therapies in cancer.

In conclusion, the altered synthesis and consumption of NAD^+^ in GBM represents a metabolic vulnerability in GBM. Here, we have identified NQO1 to be an actionable target in the selective treatment of NQO1-expressing GBM. We observe that NQO1-expressing GBM cells, regardless of subtype, are susceptible to killing by the NQO1-activated drug β-lap which damages DNA and depletes NAD^+^ levels. We find that we can augment this NAD^+^ depletion and cell killing by inhibiting the NAD^+^ salvage pathway with the NAMPT inhibitor FK866. We propose that identifying and targeting NQO1-expressing GBM with β-lap in conjunction with NAMPT inhibitors to be a promising strategy for targeting NAD^+^ metabolism in GBM.

## Supplementary Material

Supplementary Figure 1 NAD+ depletion of naphthoquinones in U87 cells. U87 cells were exposed to naphthoquinones for 2 h and NAD+ was measured. β-lap followed by plumbagin induced concentration-dependent NAD+ depletion. Lapachol had no effect on NAD+ levels. Values are mean ± SD of n≥3. 

Supplementary Figure 2 β-lap toxicity in NHA compared to U87 cells. A. β-lap has limited toxicity to NHA. NHA cells were treated with a titration of β-lap for 2 h and cells were counted by trypan-blue exclusion assay 24 h after exposure. B. β-lap is selective for NQO1-expressing U87 cells when grown in co-culture with NHA. U87 cells were fluorescently labeled with CFSE before seeded in a 1:1 ratio with unlabeled NHA in DMEM. Cells were treated with vehicle control or β-lap for 2 h. 72 h after β-lap exposure, there was significant loss of U87 cells compared to NHA. Values are mean ± SD of n=3, ANOVA and Dunnett’s Multiple Comparison Test, ***p<0.001.  

Supplementary Figure 3 β-lap induces cell death 24 h after initial exposure. A. U87 cells exposed to 2.5 and 5 μM β-lap. Cell count relative to untreated control was determined both immediately after β-lap exposure and 24 h after exposure. B. Measurements in GBM117. C. Measurements in GBM10. D. Measurements in GBM39. Values are mean ± SD of n=3. Statistical significance determined by ANOVA with Dunnett’s. *p<0.05, **p<0.01, ****p<0.0001.

Supplementary Figure 4 NAD+ synthesis pathways in PDX GBM. NQO1-expressing PDX GBM cells were treated with FK866 (10 nM) and 500 µM of each NAD+ precursor: quinolinic acid (QA), nicotinamide (NAM), nicotinic acid (NA), nicotinamide riboside (NR), or nicotinamide mononucleotide (NMN). After 24 h, cells were collected for NAD+ measurement by colorimetry assay. A. NA, NR, and NMN rescue NAD+ in GBM39 cells. NAD+ precursors NAM, NA, NR, and NMN all increased NAD+ levels in untreated GBM39 cells (black bars). FK866 depletes NAD+ levels and supplementation with 500 µM NA, NR, and NMN rescues NAD+ depletion. B-C. NR and NMN rescue NAD+ in GBM10 (B) and GBM117 (C). FK866 depletes NAD+ levels in GBM10 and 117. Supplementation with 500 µM NR and NMN rescues this depletion. QA and NA have no significant effect on NAD+ levels. Values are mean ± SD of n=3. Statistical significance determined by ANOVA with Tukey’s Multiple Comparisons. *p<0.05, **p<0.01, ***p<0.001,****p<0.0001.

Supplementary Figure 5 FK866 treatment before or after β-lap exposure does not change β-lap toxicity in U87 cells. U87 cells were exposed to 10 nM FK866 for 24 h before β-lap exposure or 24 h after β-lap exposure. These exposure conditions did not change β-lap toxicity. Values are mean ± SD of n=3.

Supplementary Figure 6 Clonal assay of U87 and NHA cells treated with β-lap and FK866. Cells were treated with a titration of β-lap in the presence or absence of 10 nM FK866 in the same manner as in Figure 5D. Colonies were counted 2 weeks post exposure. A. U87 sensitivity by clonogenic assay. U87 cells are sensitive to β-lap and addition of FK866 increases this sensitivity. B. NHA sensitivity by clonogenic assay. NHA cells are resistant to β-lap exposure and addition of FK866 does not change NHA sensitivity to β-lap. Values are mean ± SD of n=3. *p<0.05, ****p<0.0001 between 0 and 10 nM FK866 groups. #p<0.05, ####p<0.0001 compared to untreated group (0 β-lap, 0 FK866). Statistical significance determined by one-way ANOVA with Sidak multiple comparison tests.

Supplementary Figure 7 Effects of FK866 and β-lap on cell cycle. Distribution of cell cycle in U87 cells under various treatment conditions. U87 cells were treated with either (A) vehicle control (DMSO), (B) 5 μM β-lap, (C) 10 nM FK866, or (D) 5 μM β-lap with 10 nM FK866. The exposure timeline was the same as used in β-lap manuscript. Representative histograms of n=1, were repeated in triplicate.

Supplementary Figure 8 Combination of β-lap and FK866 induces rapid toxicity without caspase activation. U87 cells were treated with β-lap and combination of β-lap and FK866 in the same manner as in Figure 5D. Cells were then collected and number of cells evaluated by trypan-blue exclusion assay and caspase-3/7 activity measured by cleavage of the fluorogenic AC-DEVD-AMC substrate. Caspase-3/7 activity/μg protein was normalized to untreated control (arbitrary units, A.U.). A. Cell count after β-lap exposure. Combination treatment induced more rapid cell-death at 12 h. B. Caspase activity after β-lap exposure. β-lap induced significant caspase activity but combination did not compared to control. C. Caspase activity in FK866 treated cells. U87 cells were also treated with 10 nM FK866 and 1 µM staurosporine (STS), an inducer of apoptosis and positive control, for 24 h. Values are mean ± SD of n=3, One-way ANOVA with Tukey’s. *p<0.05, **p<0.01, ***p<0.001, ****p<0.0001.

Supplementary Figure 9 Expression levels of NQO1 and NAMPT across human tissue. Gene expression data of NQO1 and NAMPT across tissue samples from GTEx were compared. Dashed line represents the level of NQO1 and NAMPT expression in GBM for comparison.

## Data Availability

Data generated in this study are available within the article, Supplementary Data, and upon request to the corresponding author. Gene expression data analyzed in this study from TCGA (http://cancergenome.nih.gov/) and GTEx were accessed through the UCSC Xena platform (https://xenabrowser.net).
