# Supplementary figures and images for: Targeting the NAD+ Salvage Pathway Blocks Metabolic Recovery and Enhances β-Lapachone Toxicity in NQO1-Expressing Glioblastoma Cells
Source: Cancer Res Commun. 2026 Aug 3;6(8):1836–49. doi: 10.1158/2767-9764.CRC-26-0275 (PMC13430001; doi:10.1158/2767-9764.CRC-26-0275)

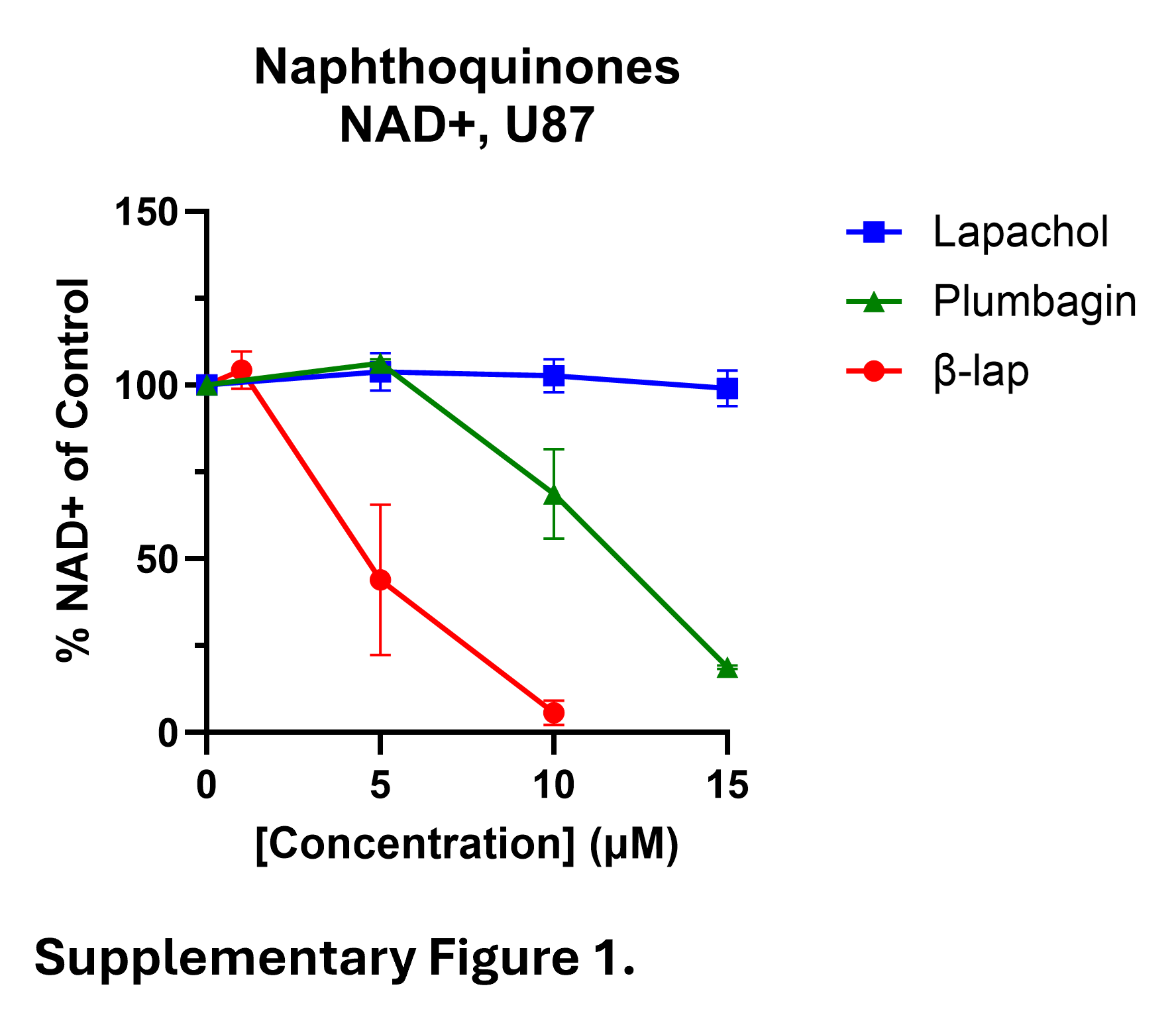

Supplement: Supplementary Figure 1 — NAD+ depletion of naphthoquinones in U87 cells. U87 cells were exposed to naphthoquinones for 2 h and NAD+ was measured. β-lap followed by plumbagin induced concentration-dependent NAD+ depletion. Lapachol had no effect on NAD+ levels. Values are mean ± SD of n≥3. [file crc-26-0275_supplementary_figure_1_suppsf1.png]

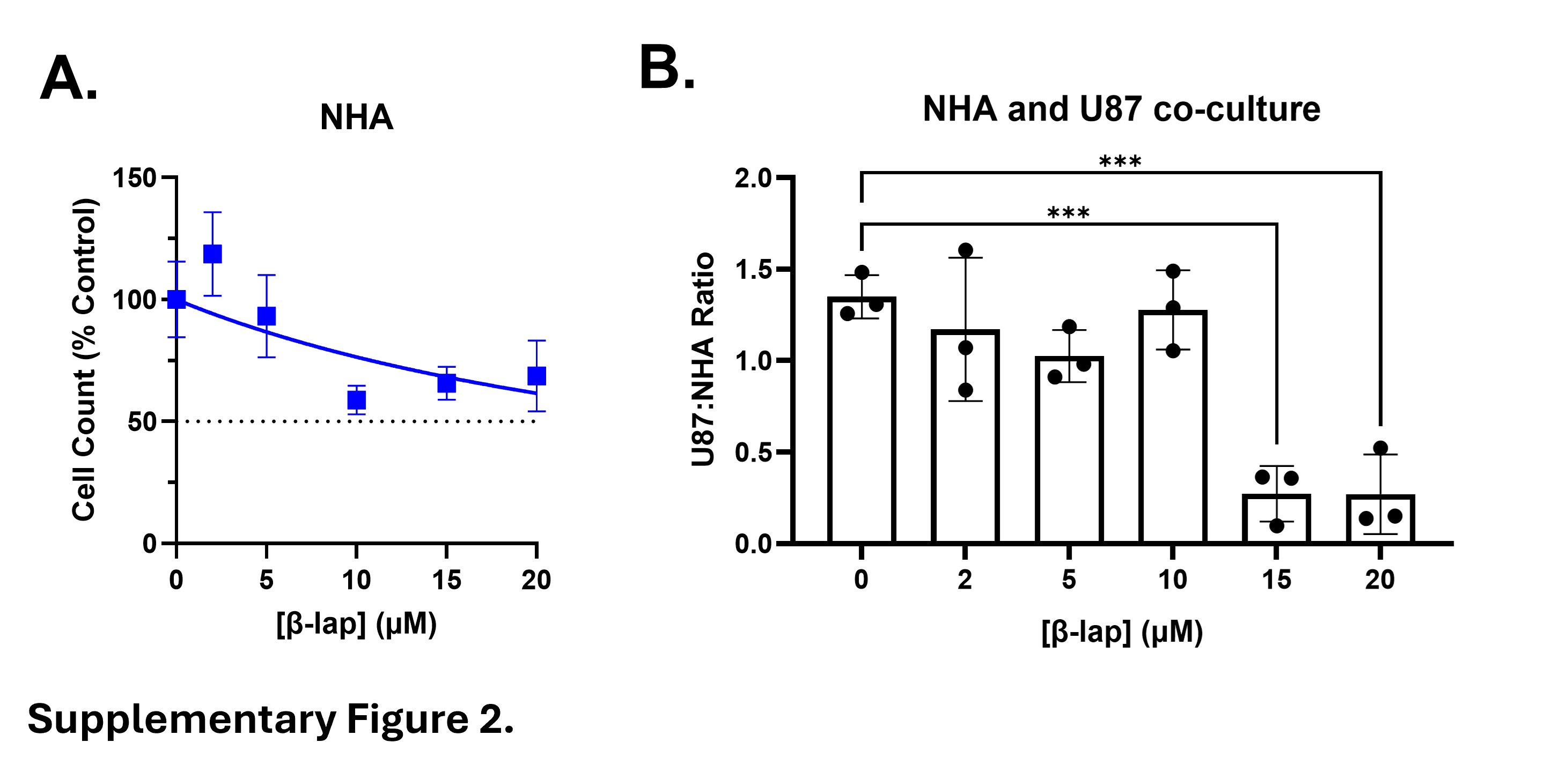

Supplement: Supplementary Figure 2 — β-lap toxicity in NHA compared to U87 cells. A. β-lap has limited toxicity to NHA. NHA cells were treated with a titration of β-lap for 2 h and cells were counted by trypan-blue exclusion assay 24 h after exposure. B. β-lap is selective for NQO1-expressing U87 cells when grown in co-culture with NHA. U87 cells were fluorescently labeled with CFSE before seeded in a 1:1 ratio with unlabeled NHA in DMEM. Cells were treated with vehicle control or β-lap for 2 h. 72 h after β-lap exposure, there was significant loss of U87 cells compared to NHA. Values are mean ± SD of n=3, ANOVA and Dunnett’s Multiple Comparison Test, ***p<0.001. [file crc-26-0275_supplementary_figure_2_suppsf2.png]

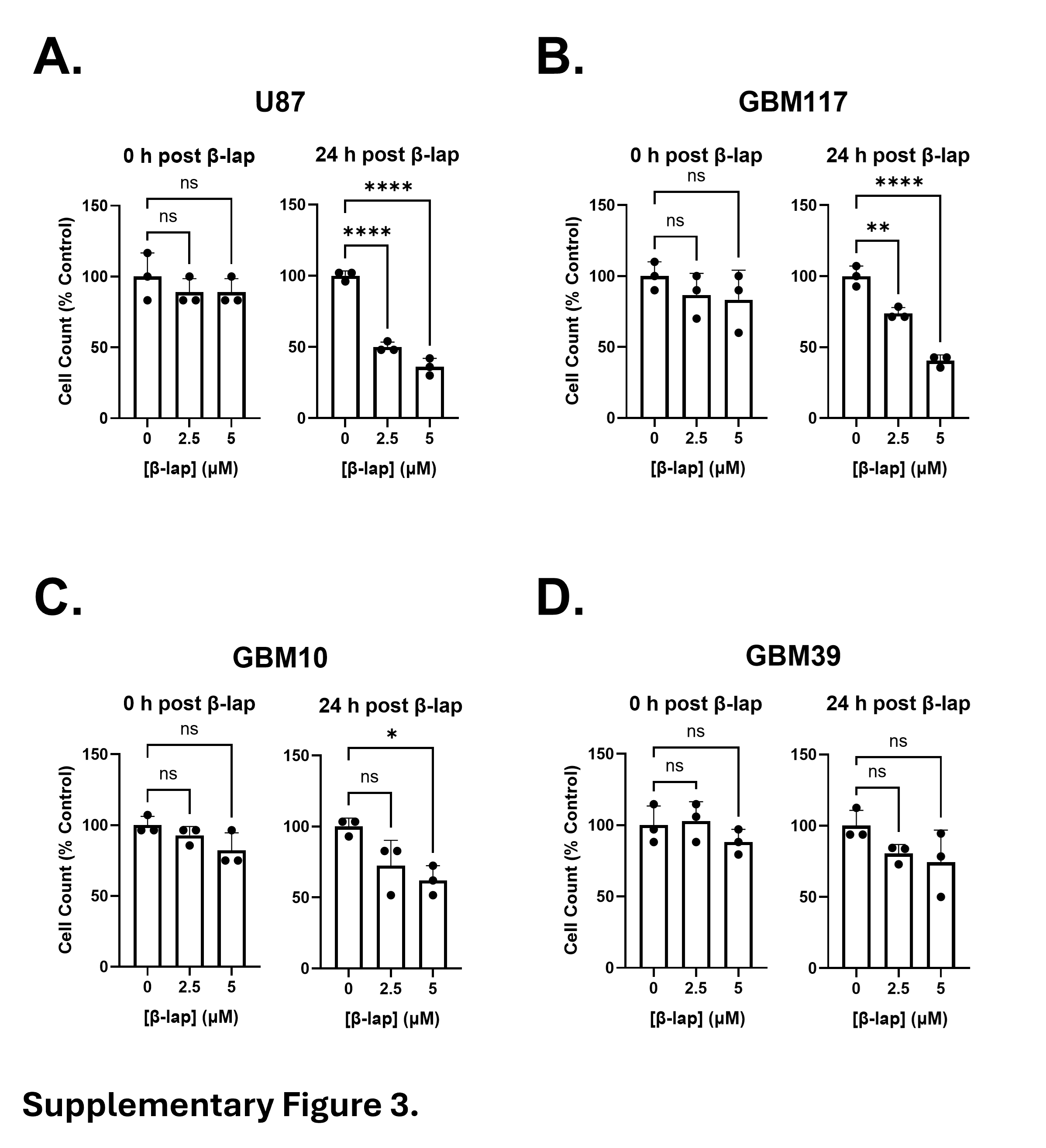

Supplement: Supplementary Figure 3 — β-lap induces cell death 24 h after initial exposure. A. U87 cells exposed to 2.5 and 5 μM β-lap. Cell count relative to untreated control was determined both immediately after β-lap exposure and 24 h after exposure. B. Measurements in GBM117. C. Measurements in GBM10. D. Measurements in GBM39. Values are mean ± SD of n=3. Statistical significance determined by ANOVA with Dunnett’s. *p<0.05, **p<0.01, ****p<0.0001. [file crc-26-0275_supplementary_figure_3_suppsf3.png]

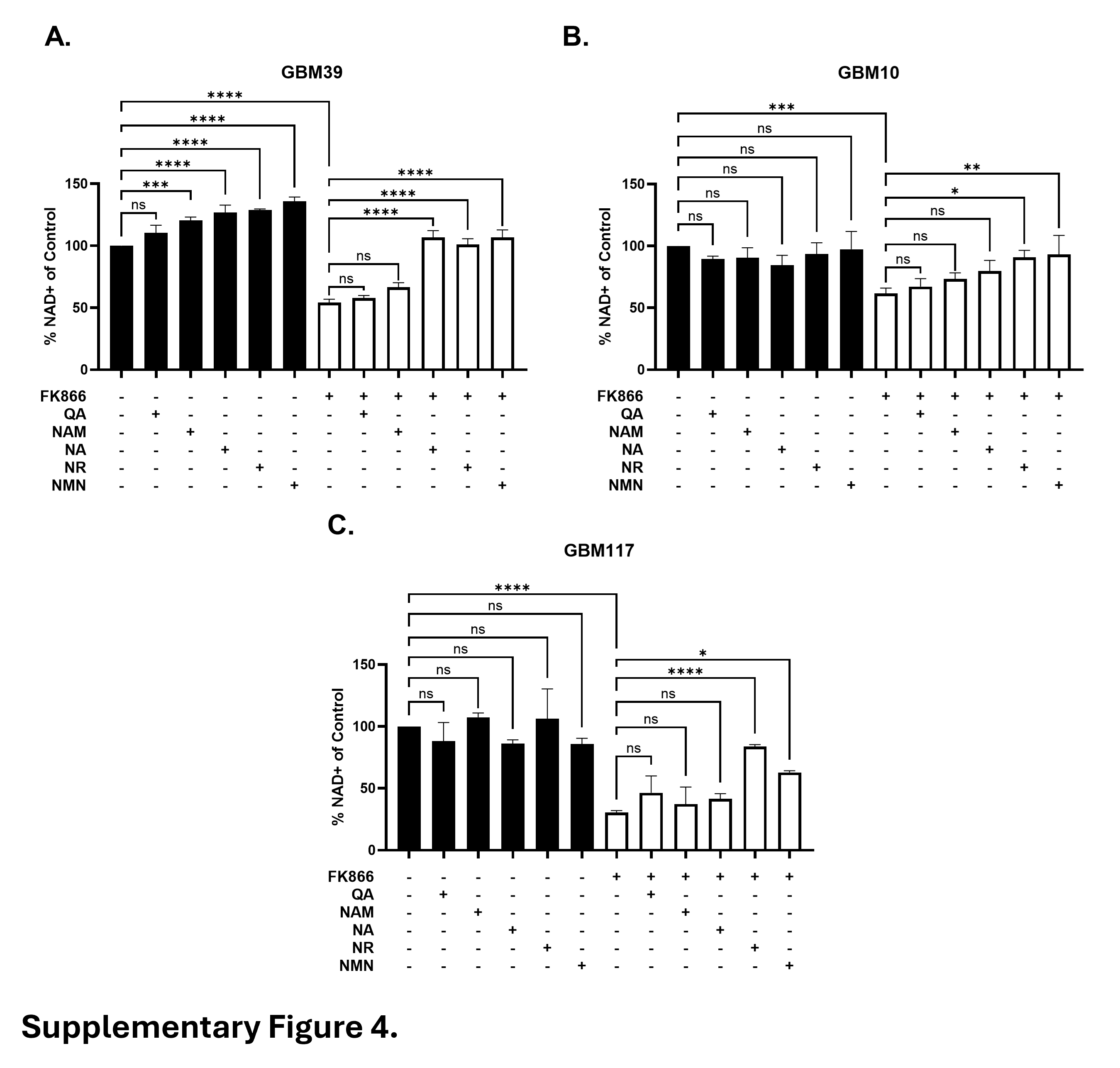

Supplement: Supplementary Figure 4 — NAD+ synthesis pathways in PDX GBM. NQO1-expressing PDX GBM cells were treated with FK866 (10 nM) and 500 µM of each NAD+ precursor: quinolinic acid (QA), nicotinamide (NAM), nicotinic acid (NA), nicotinamide riboside (NR), or nicotinamide mononucleotide (NMN). After 24 h, cells were collected for NAD+ measurement by colorimetry assay. A. NA, NR, and NMN rescue NAD+ in GBM39 cells. NAD+ precursors NAM, NA, NR, and NMN all increased NAD+ levels in untreated GBM39 cells (black bars). FK866 depletes NAD+ levels and supplementation with 500 µM NA, NR, and NMN rescues NAD+ depletion. B-C. NR and NMN rescue NAD+ in GBM10 (B) and GBM117 (C). FK866 depletes NAD+ levels in GBM10 and 117. Supplementation with 500 µM NR and NMN rescues this depletion. QA and NA have no significant effect on NAD+ levels. Values are mean ± SD of n=3. Statistical significance determined by ANOVA with Tukey’s Multiple Comparisons. *p<0.05, **p<0.01, ***p<0.001,****p<0.0001. [file crc-26-0275_supplementary_figure_4_suppsf4.png]

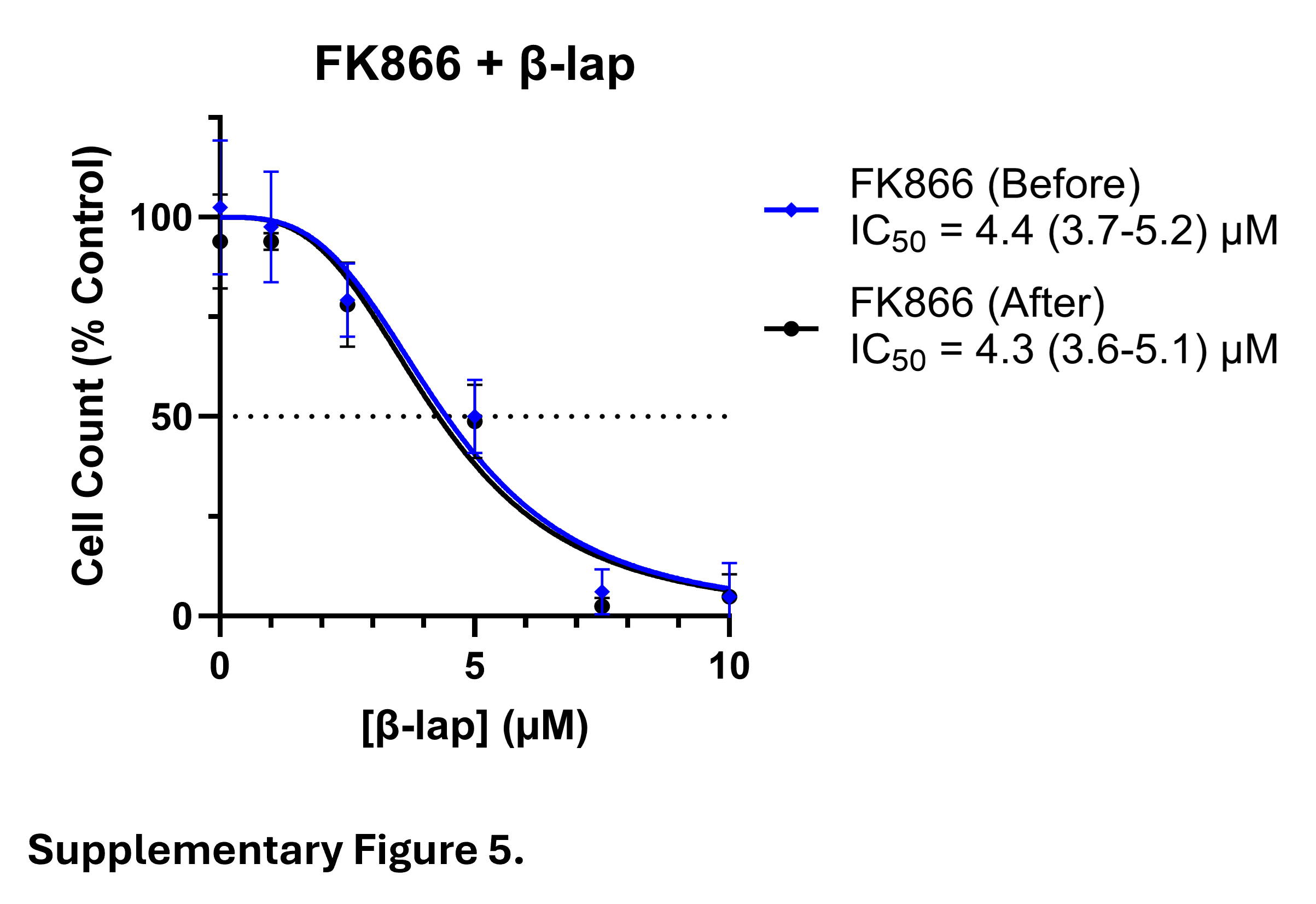

Supplement: Supplementary Figure 5 — FK866 treatment before or after β-lap exposure does not change β-lap toxicity in U87 cells. U87 cells were exposed to 10 nM FK866 for 24 h before β-lap exposure or 24 h after β-lap exposure. These exposure conditions did not change β-lap toxicity. Values are mean ± SD of n=3. [file crc-26-0275_supplementary_figure_5_suppsf5.png]

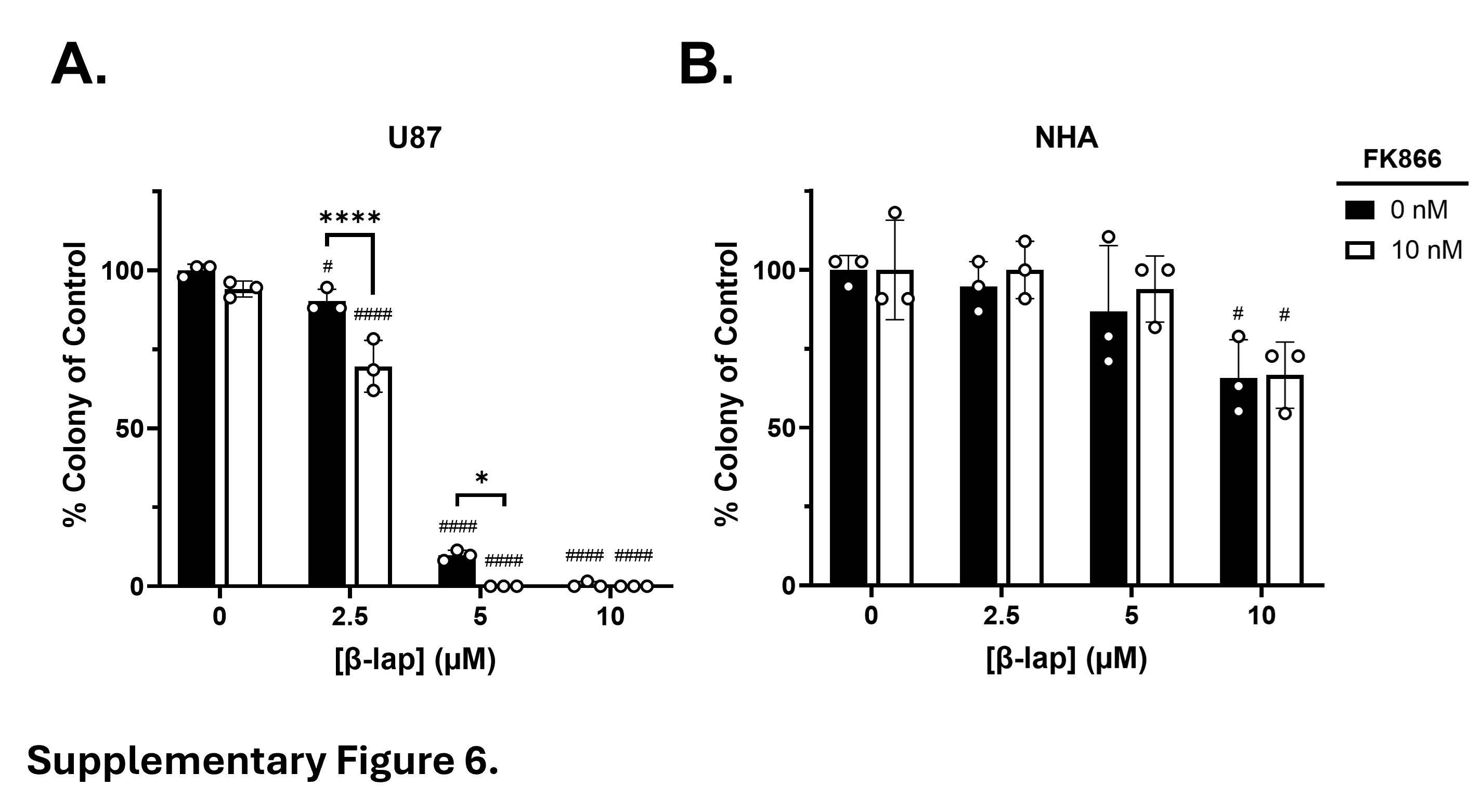

Supplement: Supplementary Figure 6 — Clonal assay of U87 and NHA cells treated with β-lap and FK866. Cells were treated with a titration of β-lap in the presence or absence of 10 nM FK866 in the same manner as in Figure 5D. Colonies were counted 2 weeks post exposure. A. U87 sensitivity by clonogenic assay. U87 cells are sensitive to β-lap and addition of FK866 increases this sensitivity. B. NHA sensitivity by clonogenic assay. NHA cells are resistant to β-lap exposure and addition of FK866 does not change NHA sensitivity to β-lap. Values are mean ± SD of n=3. *p<0.05, ****p<0.0001 between 0 and 10 nM FK866 groups. #p<0.05, ####p<0.0001 compared to untreated group (0 β-lap, 0 FK866). Statistical significance determined by one-way ANOVA with Sidak multiple comparison tests. [file crc-26-0275_supplementary_figure_6_suppsf6.png]

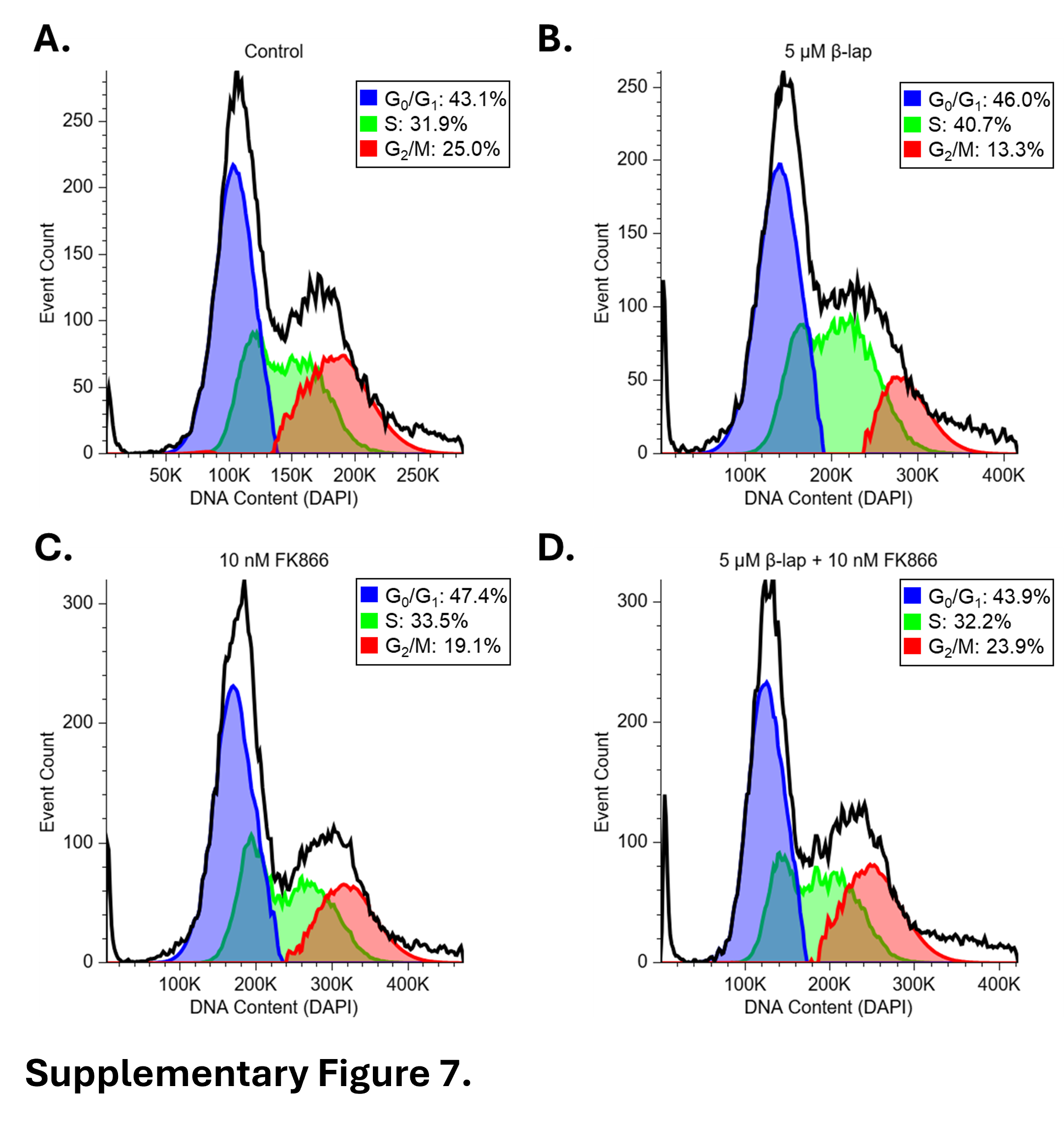

Supplement: Supplementary Figure 7 — Effects of FK866 and β-lap on cell cycle. Distribution of cell cycle in U87 cells under various treatment conditions. U87 cells were treated with either (A) vehicle control (DMSO), (B) 5 μM β-lap, (C) 10 nM FK866, or (D) 5 μM β-lap with 10 nM FK866. The exposure timeline was the same as used in β-lap manuscript. Representative histograms of n=1, were repeated in triplicate. [file crc-26-0275_supplementary_figure_7_suppsf7.png]

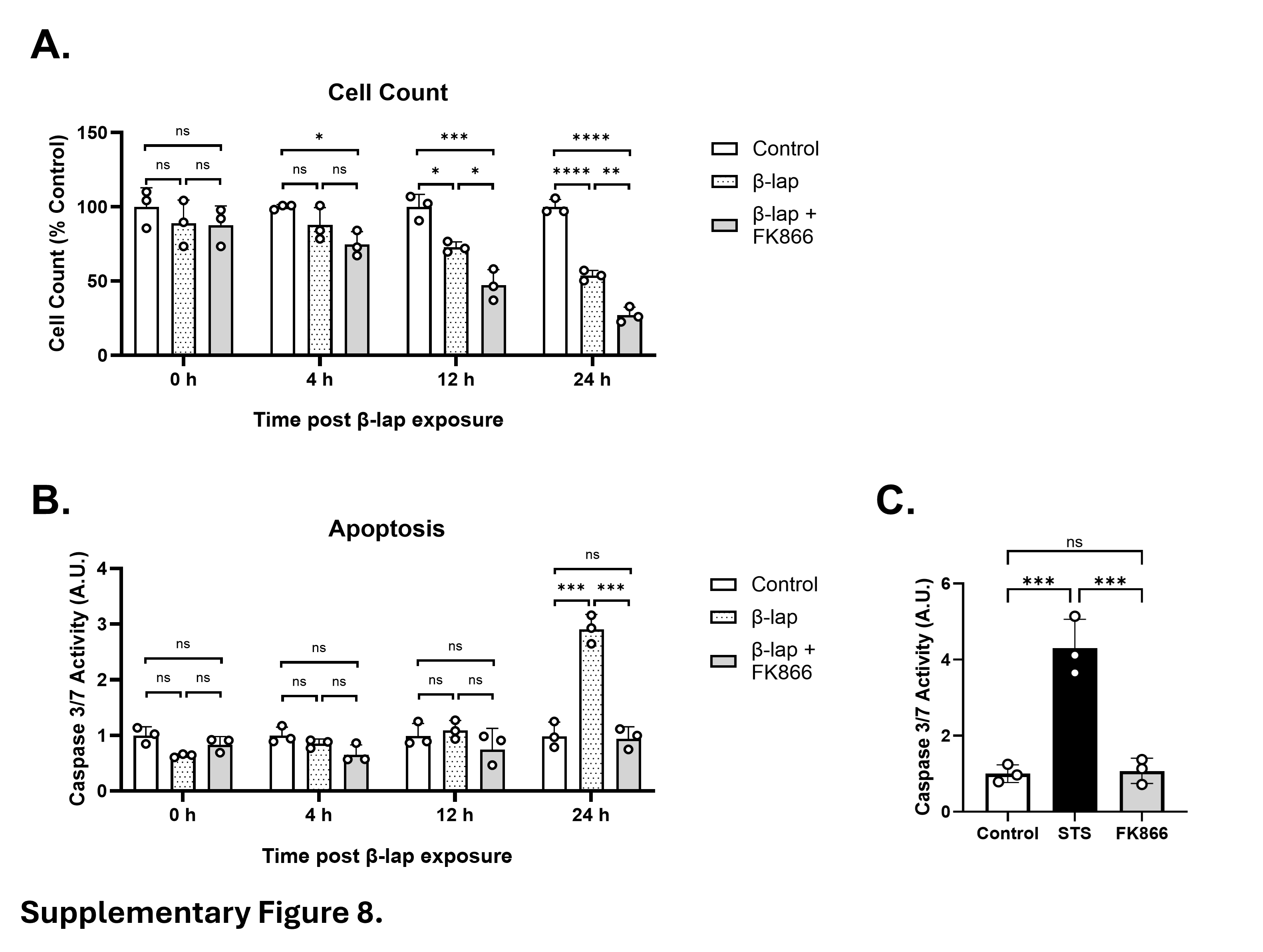

Supplement: Supplementary Figure 8 — Combination of β-lap and FK866 induces rapid toxicity without caspase activation. U87 cells were treated with β-lap and combination of β-lap and FK866 in the same manner as in Figure 5D. Cells were then collected and number of cells evaluated by trypan-blue exclusion assay and caspase-3/7 activity measured by cleavage of the fluorogenic AC-DEVD-AMC substrate. Caspase-3/7 activity/μg protein was normalized to untreated control (arbitrary units, A.U.). A. Cell count after β-lap exposure. Combination treatment induced more rapid cell-death at 12 h. B. Caspase activity after β-lap exposure. β-lap induced significant caspase activity but combination did not compared to control. C. Caspase activity in FK866 treated cells. U87 cells were also treated with 10 nM FK866 and 1 µM staurosporine (STS), an inducer of apoptosis and positive control, for 24 h. Values are mean ± SD of n=3, One-way ANOVA with Tukey’s. *p<0.05, **p<0.01, ***p<0.001, ****p<0.0001. [file crc-26-0275_supplementary_figure_8_suppsf8.png]

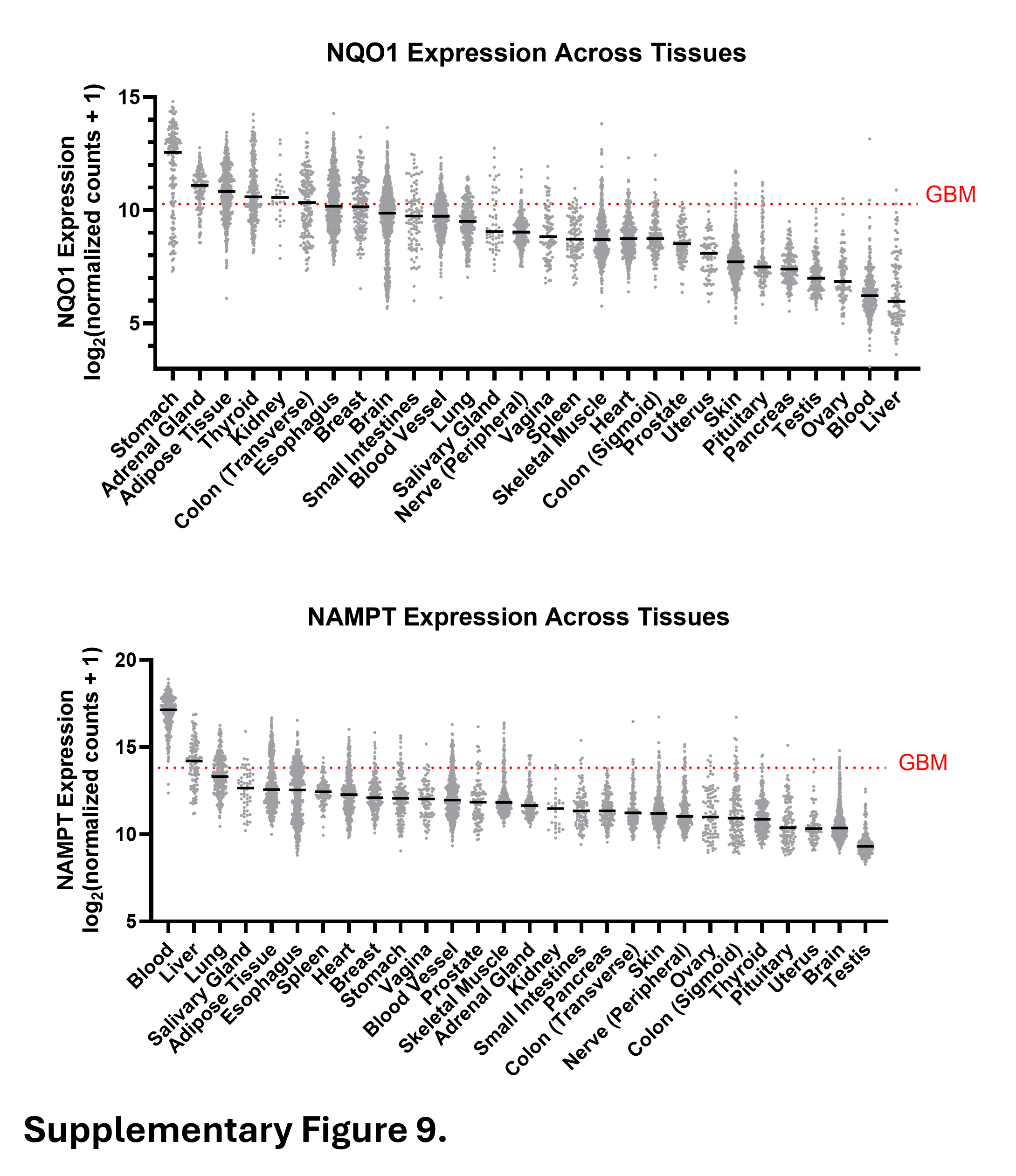

Supplement: Supplementary Figure 9 — Expression levels of NQO1 and NAMPT across human tissue. Gene expression data of NQO1 and NAMPT across tissue samples from GTEx were compared. Dashed line represents the level of NQO1 and NAMPT expression in GBM for comparison. [file crc-26-0275_supplementary_figure_9_suppsf9.png]
